# Structure–Property Relationships in Bioactive Poly (Vinyl Alcohol) Films Incorporating Natural Phenolic Additives for Sustainable Active Packaging

**DOI:** 10.3390/polym18202519

**Published:** 2026-10-14

**Authors:** Lucica Maria Pop, Lidija Jakobek, Camelia Nicula, Alexandra Maria Uivarasan, Zorica Vosgan, Monica Liliana Marian, Goran Drazic, Klara Magyari, Marieta Muresan-Pop, Ioan Botiz, Milica Todea, Lucian Baia, Cristina Mihali, Claudia Butean, Lucia Mihalescu, Oana Mare-Rosca, Monica Tarau, Ionela Traistaru Marcu, Adrian Matei Cosma, Doina Boitor Hontau, Anca Peter

**Affiliations:** 1 Faculty of Sciences, Technical University of Cluj Napoca, Victoriei 76, 430072 Baia Mare, Romania; 2 Faculty of Food Technology, Josip Juraj Strossmayer University of Osijek, Franje Kuhača 18, 31000 Osijek, Croatia; 3 Department of Materials Chemistry, National Institute of Chemistry, Hajdrihova 19, P.O. Box 660, SI-1001 Ljubljana, Slovenia; 4 Nanostructured Materials and Bio-Nano-Interface Center, Interdisciplinary Research Institute on Bio-Nano-Sciences, Babes-Bolyai University, 42, Treboniu Laurian, 400271 Cluj-Napoca, Romania; 5 Faculty of Physics, Babes-Bolyai University, M. Kogălniceanu 1, 400084 Cluj-Napoca, Romania; 6 Institute for Research-Development-Innovation in Applied Natural Sciences, Babes-Bolyai University, Fântânele 30, 400327 Cluj-Napoca, Romania; 7 Faculty of Nursing and Health Sciences, Iuliu Hațieganu University of Medicine and Pharmacy, Victor Babeș 8, 400012 Cluj-Napoca, Romania

**Keywords:** polyvinyl alcohol, polyphenols, bioactive packaging, characterization, structure, release behavior, antioxidant activity, antimicrobial activity, functional packaging, food packaging

## Abstract

This study aimed to investigate bioactive poly (vinyl alcohol) (PVA) films incorporating three chemically distinct naturally phenolic additives—aronia extract (A), apple peel extract (AP), and rutin (R)—as individual functional modifiers for active packaging applications. Here, we studied how the chemical nature of these phenolic compounds influenced the physicochemical characteristics, structure, release behavior, and functional properties of the PVA matrix. The FTIR, SEM, AFM and XPS analyses revealed intermolecular interactions between the hydroxyl groups from the PVA matrix and the hydroxyl and carboxyl groups from phenolic compounds, a high degree of miscibility between them, as well as uniform dispersion of polyphenols in the PVA matrix. XRD analysis indicated the ability of phenolic compounds to change the PVA film’s crystallinity. The films showed very low permeability to castor oil and the UV-Vis light barrier property of the film modified with aronia extract was 47% higher as compared to the reference film. The phenolic compounds improved the antioxidant activity of the reference film by about 90% and inhibited the growth of Gram-positive *Staphylococcus aureus*. The modified films proved their functionality by keeping the quality of the butter packed for 43 days, demonstrating their potential as active packaging materials.

## 1. Introduction

The development of bioactive packaging systems based on biodegradable materials is a priority for industry and academic researchers, due to important environmental problems and resource depletion caused by traditional plastic packaging [1]. Biodegradable packaging materials offer eco-friendly, sustainable, and cost-effective alternatives for food packaging. Significant progress has been made in developing biodegradable packaging materials. These materials can be derived from natural and renewable sources such as polysaccharides or proteins [2,3], which are cost-effective and readily available. Additionally, biodegradable synthetic polymers such as polylactic acid [4], polyhydroxyalkanoates [5], poly (butylene adipate terephthalate) [6], or polyvinyl alcohol [7] are widely used in various industries, including food packaging applications, due to their biocompatibility and biodegradability [8,9].

Microbial development along with lipid oxidation are the main factors that cause degradation of a wide range of foods, such as meat, milk, fish or fatty foods (i.e., nuts, oils or sauces). Alteration of food causes loss of nutritional quality and food safety and can also lead to the formation of toxic substances such as aldehydes [10]. Combating food waste and environmental concerns can be achieved by adding various bioactive natural compounds to biodegradable packaging materials. For example, various antioxidants with extensive antimicrobial activity have been used, such as grape fruit peel extract [11], apple phenols [2], yerba mate extract [12], grapefruit seed and aronia extract [13], broccoli sprout seed extract [14], or different essential oils, such cinnamon essential oil [15] and oregano essential oil [16]. Active food packaging incorporates various functional agents that confer additional attributes to the packaging systems, including antioxidant and antimicrobial activity, ethylene or moisture absorption, oxygen barrier, etc., which is essential for preserving the quality and prolonging the shelf life of food [17].

PVA is an excellent candidate for obtaining biodegradable packaging materials due to its non-toxicity, excellent film forming ability, as well as optical, thermal, mechanical, and high gas barrier properties. PVA is a synthetic semicrystalline polymer that has a skeleton made up of a long carbon chain and hydroxyl as functional group. It is obtained through the partial or full hydrolysis of polyvinyl acetate. PVA is water-soluble and insoluble in organic solvents, and is colorless, non-toxic, biocompatible and biodegradable [18,19]. PVA is a polar biopolymer, and the abundance of hydroxyl groups facilitates its interaction with other additives through hydrogen bonds. To broaden the scope, preserve food quality and safety and extend the shelf life of food, sustained efforts are being made to increase the performance of the PVA-based films. To improve antimicrobial and antioxidant properties of packaging materials, various natural bioactive agents have been reported, such as essential oils, organic acids, various plant or fruit extracts, fatty acids or agricultural residues, etc., which have been incorporated in PVA-based packaging film, thus obtaining bioactive films [12,20].

Polyphenols extracted from various sources exhibit antioxidant, antibacterial and anti-inflammatory properties. Polyphenols are frequently found in food packaging materials as natural antioxidants; their presence in the material also contributes to the cross-linking of the polymer structure, thus also improving the functional properties of packaging films [2]. Due to the hydrophilic character of PVA, it can interact with phenolic acids through inter- and intramolecular hydrogen bonds, creating a much more compact network and thus improving the performance of the film. Antioxidants found in fruits or bio-waste reduce oxidative stress by capturing free radicals and inhibiting enzymes [19].

Aronia (*Aronia melanocarpa*), also known as black aronia, is regarded as one of the richest sources of polyphenols among all berries and even among many other plant-based foods [21]. Aronia possesses strong antioxidant activity due to the presence in its structure of phenolic compounds, such as anthocyanins and procyanidins, responsible for the astringent taste of aronia; phenolic acids; and flavonols, mainly derivatives of quercetin and epicatechin [13,22]. Aronia has been incorporated into various biopolymer films to confer antioxidant and antimicrobial properties. Because of the sensitivity of natural dyes to pH, thus changing the anthocyanin structure, aronia is an ideal component to produce intelligent packaging allowing for the monitoring of the freshness of food. Thus, Oun et al. [13] incorporated various functional agents, namely aronia (A) extract, grapefruit seed extract (G) and cellulose nanoparticles (CNC), into the PVA and chitosan bio-composite, to obtain multifunctional films. The aronia extract gave the film high thermal stability, antioxidant activity and sensitivity to a wide pH range as well as moderate antimicrobial activity. CNCs improved tensile strength and water vapor permeability, and grapefruit seed extract provided flexibility to the film, improved the antimicrobial and antioxidant activity of the film, as well as the opacity. Long et al. [23] added aronia extract in various mass proportions (4–16%) into the PVA and chitosan film (CS) to improve the physicochemical and functional properties of the film. The PVA/CS film containing 8% aronia extract obtained the best results in terms of the antioxidant and antimicrobial activity, miscibility, UV protection barriers, water vapor permeability, and mechanical properties, as well as visible color changes when pH changes during shrimp packaging. Intelligent packaging films based on cassava starch/polyvinyl alcohol with the addition of aronia extract were obtained by Wu et al. [24] for monitoring milk freshness. The modified films showed improved mechanical strength, permeability to water vapor and UV-Vis light, and demonstrated high sensitivity to pH changes during milk monitoring, with color changes more noticeable as the amount of aronia extract added increased.

Apples, cultivated in abundance in over 7500 varieties, are also an important source of phenolic compounds. Electrophilic groups, which contain apple polyphenols, including phenolic hydroxyl groups, capture and neutralize free radicals, thus preventing oxidative damage. Also, the polyphenols in apples can inhibit enzymatic activity, conferring strong antioxidant properties by reducing oxidative reactions [1]. Phenolic compounds present in apples include procyanidins, quercetin glucosides, cyanidin-3-galactoside, epicatechin and catechin and chlorogenic acid. Studies have shown that procyanidins and flavonoids present in apples have antibacterial, antioxidant, anticancer, immunomodulating, cardioprotective, antimicrobial, anti-inflammatory, anti-obesity, neuroprotective, antiproliferative and anti-aging properties. Apple peel, compared to pulp, contains 5 and 3.6, respectively, times more phenolic compounds, depending on the apple variety. The peel, as a valorized agri-food by-product, rich in phenolic compounds, contributes to both the functionality of the material and the sustainability of the process [25,26,27]. In the attempt to obtain food packaging with antimicrobial and antioxidant properties Riaz et al. [28] incorporated polyphenols from the apple peel. The results of the study indicated improved physical properties, namely density, solubility, thickness, opacity, moisture content, swelling ratio, as well as water vapor permeability. The films obtained also showed improved mechanical properties and thermal stability. The antimicrobial activity of the modified apple peel films was concentration-dependent and was demonstrated against four microorganisms, namely, *S. aureus*, *E. coli*, *B. cereus* and *S. typhimurium*, and the antioxidant activity of the films increased directly in proportion to the increase in polyphenols. Multifunctional nanocomposites were obtained by Van Nguyen and Lee [29] by adding apple peel extract to the PVA film reinforced with cellulose nanocrystals (CNC), with the addition of titanium dioxide (TiO_2_). The best results were obtained by PVA/CNC nanocomposites containing 5% by weight of CNC, modified with 5% by weight of TiO_2_ and 20% extract of apple peel, which improved the mechanical strength of PVA film by 49.9% and water vapor permeability by 36.6%, giving the film excellent UV barrier properties and high antibacterial and antioxidant properties. The obtained nanocomposites proved their effectiveness in extending the shelf life of cherry tomatoes and slices of fresh potatoes.

The rutin is a flavonoid glycoside that has antioxidant, antimicrobial, anti-inflammatory, anti-allergic, antifungal, anticancer, cardioprotective, and neuroprotective properties. It can be found under various names, such as quercetin-3-rutinoside, rutoside or vitamin P. Rutin is present in over 70 plant species, but buckwheat is the predominant source. It can also be extracted from aronia, nettle, fresh olives, apples, citrus fruits, etc. Rutin takes the form of yellow crystalline powder and has a bitter taste. The rutin is soluble in ethanol, methanol and dimethyl sulfoxide and is insoluble in ether and chloroform. The rutin has low bioavailability due to low water solubility, lipo-solubility and stability. The ability of the rutin to eliminate free radicals and reactive oxygen species has been demonstrated in the literature, thus preventing lipid peroxidation. The rutin has a protective role against damage caused by oxidative stress. The antioxidant activity of the rutin consists in improving the activity of endogenous enzymes, such as catalase, superoxide dismutase and glutathione dismutase [30,31,32]. The rutin has been used as a bioactive compound in the production of various food packaging materials, with the aim of preserving quality and extending shelf life. Roy and Rhim [15], for instance, developed gelatin and chitosan films modified with cinnamon essential oil (CEO) and rutin. Bioactive compounds have demonstrated their compatibility with the polymer matrix, obtaining transparent films with barrier properties against UV light and water vapor, as well as better mechanical and thermal properties. The addition of CEO and rutin resulted in films with high antioxidant and antibacterial properties. The rutin showed greater free radical scavenging by donating electrons or hydrogen due to hydroxyl groups present in its structure. Antimicrobial activity has been demonstrated against *E. coli* and *L. monocytogenes*, with CEO showing stronger activity than rutin, but the authors attributed this to twice as much CEO as rutin (2% CEO and 1% rutin). The authors of this study recommend functional films obtained as potential packaging materials to prevent lipid oxidation and to provide food safety. Also, rutin induced chitosan/poly (vinyl alcohol) bioactive films have been developed by Narasagoudr et al. [33] to improve the physical–chemical and functional properties of the films. Mechanical and thermal properties, barrier properties to water vapor, oxygen and UV light have been improved and there was a decrease in the water solubility of the films. Compatibility between the film components has been demonstrated by SEM and FTIR analyses by forming strong intermolecular bonds between functional groups. The bioactive films obtained indicated strong antimicrobial activity, inhibiting the growth of *E. coli* and *S. aureus* bacteria, and demonstrated biodegradability faster than reference films.

The principal contribution of this work is not only the development of bioactive PVA films incorporating three chemically distinct naturally derived phenolic additives—aronia extract, apple peel extract, and rutin—but also the establishment of clear composition–structure–property relationships through a comprehensive multiscale characterization approach. The phenolic composition of the natural extracts was first determined by HPLC, providing a chemical basis for interpreting the behavior of the modified polymer systems. The incorporation and distribution of the bioactive compounds were subsequently investigated using FTIR spectroscopy, SEM–EDXS, XPS, AFM, and X-ray diffraction. Thermal stability was further assessed by TG/DTA analysis, and the mechanism of the release of phenolic compounds from the films was evaluated to establish the relationship between polymer structure and the availability of the active molecules. Their potential applicability as functional materials usable in the food industry was tested by assessing the quality of cow butter monitored for 77 days. Through this approach, the research contributes to the expansion of knowledge on the development of active PVA-based packaging, providing new perspectives for the exploitation of natural resources and food by-products in packaging applications.

## 2. Materials and Methods

### 2.1. Materials

Poly (vinyl alcohol) Mowiol^®^ 28–99, M_w_ = 145,000 g·mol^−1^ was purchased from Sigma-Aldrich^®^ (Merck, Darmstadt, Germany). Glycerin (Gly) and citric acid monohydrate (CA) was provided by Chemical Company, Romania. Orto-phosphoric acid (85% HPLC grade) was from Fluka (Buchs, Switzerland). Methanol (HPLC grade) was from J.T. Baker (Gliwice, Poland). Sodium carbonate was purchased from Kemika (Zagreb, Croatia). Folin–Ciocalteu’s phenol reagent was purchased from Sigma-Aldrich^®^ (Merck, Germany). Ethanol 96% was purchased from Chemical Company, Romania. Rutin powder was purchased from Now Foods, Bloomingdale, IL, USA. Apples (Red Delicious) and aronia were purchased from a local producer in Baia Mare, Romania.

### 2.2. Development of Films—Preparation Recipes

#### 2.2.1. Preparation of the Aronia, Apple Peel and Rutin Extracts

The apple peels (AP) and aronia (A) fruits were dried in the oven at 40 °C for 48 h, after which they were ground with a coffee grinder and kept in the freezer until use. To prepare the apple peel and aronia extracts, 10 g of dried apple peel and aronia, respectively, were dissolved in 100 mL distilled water and were homogenized in a shaking incubator type LBX Instruments (Grupo Labbox, Premià de Dalt, Barcelona, Spain) WBO for 24 h. The extracts thus obtained were initially filtered and centrifuged by using a Hettich EBA 20C (Andreas Hettich GmbH, Tuttlingen, Germany), for 10 min at 6000 rotations per minute to remove impurities. The rutin (R) solution was obtained by dissolving 0.1 g rutin powder in 5 mL of ethanol solution for 2 h, using a vortex (Grant Bio, Cambridgeshire, England), the mixture obtained being centrifuged for 10 min at 6000 rotations per minute. The total polyphenol content (TPC) of the extracts was 1.85 mg GAE/g film for AP, 5.51 mg GAE/g film for A and 1.28 mg GAE/g film for R.

#### 2.2.2. Preparation of Modified PVA Films with Aronia, Apple Peel Extracts and Rutin

The films were made using the solution casting method. The PVA film was prepared and noted as the reference film, termed PVA. This film was prepared by mixing 15 g of PVA granules with 0.55% (wt/*v*) CA in 300 mL distilled water, in a Berzelius glass beaker, covered with watch glass, for 3 h, at 80 °C, on a Heidolph MR Hei-End magnetic stirrer, on a water bath, until a homogeneous solution was obtained. Finally, 1.5% (*v*/*v*) Gly was added in the film forming solution and the entire amount was mixed for another 5 min. A 10 mL quantity of film-forming solution was poured into 90 mm diameter glass Petri dishes and dried for 48 h at room temperature. To obtain the modified PVA films with aronia and apple peel extract, the same procedure was used, except that distilled water was replaced with the aronia and apple peel extracts. The modified PVA film with aronia extract was noted PVA + A and the modified PVA film with apple peel extract was noted PVA + AP. The modified PVA film with rutin was prepared in the same way as the PVA-based film, but the rutin solution was added after 3 h of magnetically stirring and left to homogenize for another 5 min. The film was noted PVA + R. Three samples were prepared for each formulation.

### 2.3. Characterization of Films and Extracts

#### 2.3.1. Determination of Polyphenols by Reversed-Phase High-Performance Liquid Chromatography with Photodiode Array Detection (HPLC-PDA)

HPLC 1260 Infinity II system (Agilent technology, Santa Clara, CA, USA) containing a quaternary pump, a PDA detector and a vial sampler were used for the analysis of an aliquot of 1 mL sample from each extract. A Poroshell column 120 EC C-18, 4.6 mm × 100 mm, 2.7 μm and a Poroshell protection column 120 EC C-18 of 4.6 mm were used for separating the phenolic compounds; 0.1% H_3_PO_4_ was used as mobile phase A, and 100% HPLC-grade methanol as mobile phase B. The mobile phase flow was adjusted to 0.8 mL min^−1^, and the injection volume was 10 µL in line with the methods developed and calibrated by Jakobek et al. [34].

#### 2.3.2. Morphological Analysis by Electron Scanning Microscopy (SEM) and Elemental X-Ray Analysis with Energy Dispersion (EDXS)

The film’s morphology was determined by optical and electronic microscopy. The optical images were recorded by using a BRESSER LCD Micro Microscope (Rhede, Germany), with 40× magnification. The SEM images were recorded with a Jeol ARM 200 CF SEM Cs system (Tokyo, Japan). The samples were covered with a thin layer of carbon to enable surface conductivity. Elemental analysis was determined by energy-dispersive X-ray spectrometry (EDXS). Energy-dispersive X-ray spectrometer (SDD) was employed, specifically the Jeol Centurion model (Tokyo, Japan). The working potential was set to 80 kV. The approximate calculation of the composition was performed using the Oxford Instruments AZtec EDXS software (Version 5.1), considering the thin layer of carbon coating.

#### 2.3.3. Morphological Analysis by Atomic Force Microscopy (AFM)

All AFM images were recorded in non-contact (tapping) mode using a system from Molecular Devices and Tools for Nano Technology (NT-MDT) mounted on an Olympus IX71 optical microscope and acquired from SpeJctrum Instruments Ltd. (Limerick, Ireland). The AFM measurements were conducted by employing high-resolution Noncontact Golden Silicon probes from NT-MDT and exhibiting a tip radius of a curvature smaller than 10 nm and a tip height of about 15 ± 1 µm. All probes were also Au-coated on the detector side cantilever. The latter displayed a length of 125 ± 5 µm and a resonance frequency in the range of 187–230 kHz. Moreover, the nominal force constant of the cantilever was ranging between 1.45 and 15.1 N/m. The AFM images (256 × 256 lines) were acquired using a typical scanning speed of about 1–2 µm/s and a setpoint carefully adjusted between 9 to 12 V to maintain a very soft tapping regime.

#### 2.3.4. Fourier Transform Infrared Spectroscopy (FTIR) Analysis

The analysis was carried out using the Perkin Elmer FTIR spectrophotometer in the 4000–600 cm^−1^ range. The FTIR spectrum was obtained by overlapping 10 replicas. The resolution was 4 cm^−1^.

#### 2.3.5. X Ray Diffraction Structural Analysis (XRD)

The crystalline phases of composite films were identified using a Shimadzu X-ray diffractometer (XRD 6000, Kyoto, Japan), operated with Cu Kα radiation (λ = 1.54 Å) and a Ni filter. The diffraction patterns were recorded in 2θ range from 10° to 80° with a scan speed of 2°/min. Quantitative determination of the crystallinity index (CI, %) of the samples was determinate using OriginPro 9.0 software (OriginLab Corporation, Northampton, MA, USA) with the Peak Analyzer tool, as described in Supplementary Material Section S1 [35,36,37,38]. The relative degree of crystallinity (crystallinity index, CI) was quantitatively calculated from the resolved peak areas according to the following equation:

(1) $$\mathrm{CI}\left(\%\right)=\frac{A_{c}}{A_{c}+A_{a}}\cdot 100$$

#### 2.3.6. X-Ray Photoelectron Spectroscopy (XPS) Analysis

The XPS spectra was performed using a SPECS PHOIBOS 150 MCD system (Berlin, Germany) equipped with a monochromatic AlKα source (200 W, hν = 1486.6 eV), a hemispherical analyzer and a multichannel detector. Experiments were obtained by operating an X-ray source with a power of 200 W. The analysis chamber pressure was between 10^−9^–10^−10^ mbar range. Charge neutralization procedure was used for all samples using a flood gun component. The binding energy scale was referenced to the carbon component C1s at 284.6 eV. Survey spectra were acquired at a pass energy of 60 eV were used to determinate the elemental compositions (at %). High-resolution spectra were collected at an analyzer pass energy of 20 eV. The Shirley method was applied for background subtraction in the fitting procedure.

#### 2.3.7. Differential Thermal Analyses (DTA) and Thermogravimetry (TGA)

The evaluation of the thermostability and thermal degradation behavior of the prepared films was performed in the 30–800 °C temperature range using a thermal analyzer DTG-60H (Shimadzu, Kyoto, Japan), which simultaneously records the differential thermal analyses (DTA) curve and thermogravimetric curve (TGA). The sample was placed in an alumina crucible and covered with a perforated alumina lid, the reference material being α-alumina. The samples were heated in the 30–800 °C temperature range, with a heating rate of 10 °C/min, in a nitrogen atmosphere with a flow of 70 mL/min. TA60 software (version 2.20) was used to analyze data.

#### 2.3.8. Permeability to Water Vapor (WVP)

The gravimetric determination followed Sobral et al. [39], with adaptations for this study. Film samples were conditioned for 48 h at 25 °C and 53% relative humidity (RH). The film piece was fixed to a plastic 1 cm × 1 cm vial containing anhydrous silica gel (~3 g) and placed at 40 °C and 36% RH. The cells were weighed daily until constant mass was reached, defined as a mass difference of ≤0.0002 g between two consecutive measurements. The WVP was calculated with the formula

(2) $$\mathrm{WVP}\;\left(g/s\cdot m\cdot \mathrm{Pa}\right)=\frac{w\cdot x}{t\cdot A\cdot ∆P}$$

where w—increase in mass due to the absorption of water vapor by silica gel until at equilibrium (g).

x—material thickness (m).

A—contact surface (m^2^).

t—the analysis time required to reach equilibrium (s).

ΔP—the difference between the partial pressure of the atmosphere with silica gel and distilled water (2652 Pa at 40 °C).

#### 2.3.9. Permeability to Fat

To determine the permeability to castor oil, the Tappi–T559 [40] method was used. A quantity of 2 g castor oil was inserted into the plastic bottle, and the vial was covered with the film to be analyzed. Subsequently, the vial was turned over onto a piece of filter paper (weighed in advance) and was kept both refrigerated (4 °C) and room temperature (20 °C) for 24 h. After that, the filter paper was re-weighed, and the grease permeability was determined as the percentage of oil that penetrated the film within 24 h.

(3) $$\mathrm{Permeability to fat}\left(\%\right)=\frac{m_{f}-m_{i}}{m_{i}}\cdot 100$$

where m_f_—mass of filter paper after 24 h in storage (g).

m_i_—the mass of the filter paper at the beginning of the experiment (g).

#### 2.3.10. UV–Vis Light Barrier Property of Films

The barrier against UV-Vis radiation of the film obtained was determined using the Perkin Elmer Lambda 35 spectrometer according to the method described by Gómez-Estaca et al. [41]. The wavelength range varies between 200 and 800 nm. The light transmittance of the film was determined by the formula

(4) Light transmittance (mm−1)=Abs.  at 600 nmx 

where Abs. at 600 nm—absorbance at 600 nm.

x—film thickness (mm).

#### 2.3.11. Water Absorption (Equilibrium Water Content—EWC)

The analysis consists in monitoring the film mass before and after immersion in a phosphate buffer solution pH = 7.4, until equilibrium (~3 h) is reached at 37 °C. A piece with a known film mass is inserted into 10 mL phosphate buffer pH = 7.4 and stored at 37 °C for 3 h. Subsequently, the piece of film is removed, the excess liquid is absorbed with filter paper and weighed. The EWC has been calculated with the formula

(5) $$\mathrm{EWC}\left(\%\right)=\frac{m_{f}-m_{i}}{m_{i}}\cdot 100$$

where m_i_—the initial mass of film pieces (g).

m_f_—the final mass of the film piece (g).

#### 2.3.12. Wetting

The compatibility of the films obtained with fatty foods was tested by determining the contact angle formed by applying the film-forming solution on the surface of a piece of butter with a syringe equipped with a needle with an external diameter of 0.5 mm. The image was photographed with a Canon MP-E 65 mm f/2.8 1–5× Macro Photo Lenses, focused using a MC1 microscope-type Epi and processed using the ImageJ-win32 Software program (Version 1.53t), whose operation is based on the goniometric algorithm. Contact angles were measured at 25 ± 1 °C on both sides of the drops, at least 5 replicas being made for each and the results are presented as arithmetic means. Photographing the contact angles was done within 10 s of dropping drops to avoid absorption.

#### 2.3.13. Solubility in Food Simulants

The method consists of assessing the solubility of films in simulant A (10% ethanol solution that mimics aqueous food), simulant B (3% acetic acid solution that mimics acidic food) and simulant D1 (50% ethanol solution that mimics fatty food) by gravimetric analysis. Three pieces (2 cm^2^) were cut out of each film and weighed and were then immersed into 10 mL of simulant. The samples were left at 20 °C and 4 °C for 24 h, and were afterwards removed, dried and weighed. The solubility was calculated according to the formula:

(6) $$S\left(\%\right)=\frac{m_{i}-m_{f}}{m_{i}}\cdot 100$$

where S—solubility (%).

m_i_—the initial mass of film pieces (g).

m_f_—the final mass of film pieces (g).

#### 2.3.14. Capacity of Thermo-Welding

This property was tested using a VacSy^®^ Bag vacuum cleaner, Zepter, Romania, with automatic vacuum function.

#### 2.3.15. Tensile Strength

The tensile strength was determinate as the pressure applied to break a piece of material, with a size of 1.1 cm/3 cm, and the mechanical strength was calculated with the formula:

(7) $$\mathrm{TS}=\frac{m\cdot g}{t\cdot h}$$

where m—mass that breaks the material (kg).

g—gravitational acceleration (9.81 m/s^2^).

t—width of the material (m).

h—material thickness (m).

#### 2.3.16. Antioxidant Activity

The antioxidant activity was established based on the 2,2-diphenyl-1-pichrylphydrazil method (DPPH). The antioxidant activity was determined spectrophotometrically using the Perkin Elmer Lambda 35 spectrometer. The method was proposed by Tongnuanchan et al. [42]. A quantity of ~0.1 g dry package, cut into small pieces, was treated with 10 mL methanol and homogenized for 3 h. A volume of 3 mL methanolic extract was treated with 3 mL DPPH 0.15 mM ethanol solution (DPPH in 95% ethanol) in the dark for 30 min at room temperature. The mixture was stirred by using an orbital Heidolph stirrer. The absorbance was read at 517 nm, and the antioxidant activity was determined with the formula

(8) Antioxidant activity %=(1−A sampleA DPPH)·100

where A_sample_—sample absorbance.

A_DPPH_—blank absorbance.

#### 2.3.17. Antimicrobial Activity

The antimicrobial activity of the PVA films was tested against Gram-positive *Staphylococcus aureus* (*S. aureus*) strains isolated from goat’s milk and confirmed by catalase and coagulase assays. Isolation of *S. aureus* strains was carried out in the microbiology laboratory according to ISO 6888-1:2021 [43]. Decimal dilutions were prepared in sterile saline (1:9), followed by inoculation on Baird-Parker Agar Base supplemented with egg yolk emulsion and potassium tellurite, using the spread plate method. The plates were incubated at 37 °C for 24–48 h. Typical colonies, characterized as black, shiny and convex, often surrounded by a transparent halo, were considered presumptive *S. aureus*. The presumptive isolates were subsequently confirmed by catalase testing using 3% hydrogen peroxide and by the coagulase test using rabbit plasma; both tests yielded positive results.

An adapted disc diffusion method was used to evaluate the antimicrobial activity of the PVA films. The bacterial inoculum used for the antimicrobial assay was 2 × 10^8^ CFU/mL. The bacterial suspension was uniformly inoculated onto the surface of the appropriate agar medium using the lawn culture technique. Circular film samples with diameters of 5 mm and 20 mm were then placed on the inoculated agar surface. Following incubation at 37 °C for 48 h, the inhibition zones were evaluated by measuring the zone extending beyond the edge of the film samples.

Positive and negative controls were not included as separate controls in the original antimicrobial assay. Therefore, the PVA-based formulation without phenolic compounds was considered as the matrix control for evaluating the additional antimicrobial contribution of the phenolic compounds.

The antimicrobial activity was evaluated by measuring the inhibition zone around the PVA-based film samples. Since film discs with different diameters (5 and 20 mm) were used, the inhibition zone was determined as the distance extending from the edge of the film disc to the point at which visible microbial growth resumed. Thus, the measured value represents the width of the clear zone beyond the film edge and does not include the diameter of the film itself. The inhibition zone was measured at several points around the film disc, from the edge of the film to the point at which visible microbial growth resumed, and the mean value was calculated. This approach was used to allow for comparison of the antimicrobial activity among samples with different film disc diameters.

#### 2.3.18. The Release of Total Polyphenols from the Films Obtained

The Folin–Ciocalteu method was used for the determination of polyphenols in extracts and the polyphenols released from the films obtained. The absorbance from the blank at 765 nm was measured with a UV-Vis Spectrophotometer Shimadzu UV-1280. The total polyphenol released from films was calculated as absorbance vs. concentration using the gallic acid calibration curve and expressed as µg of gallic acid equivalents (GAEs) per g of film. For the preparation of samples, 0.5 g of each film obtained, cut into small pieces, were introduced into 3 mL 50% ethanol solution, previously held for 30 min in ultrasonic bath. A 30 µL sample aliquot was taken by pipette at 5, 15, 30, 60, and 120 min and put in test tubes with 2370 µL distilled water, 450 µL of sodium carbonate (200 g·L^−1^) and 150 µL of Folin–Ciocalteu regent. After that, 3 samples for each specific time were incubated for 30 min at 40 °C and analyzed. Gallic acid solutions (0 to 500 mg·L^−1^) were measured by the same procedure.

To elucidate the mass transport mechanism governing the release of polyphenols from the composite films and to quantitatively describe the initial burst phenomenon followed by the plateau phase, the experimental release profiles were fitted to mathematical kinetic models using OriginPro 9.0 software (OriginLab Corporation, Northampton, MA, USA). The mathematical modeling of polyphenol release kinetics is described in Supplementary Material Section S2 [44,45,46,47,48].

#### 2.3.19. Testing the Packaging Films to Reduce the Oxidation of Butter in Storage

The packaging performance of the produced films was tested by storing the cow butter with 82% fat for a period of 77 days in refrigeration conditions. The freshness and quality of the butter were assessed using physicochemical methods. To assess the ability of the developed biofilms to limit mass transfer (the exchange of moisture with the atmosphere of the storage medium), the gravimetric evolution of the food matrix system (butter with 82% fat) and the related protective film were monitored. The peroxide index (PV) was determined to assess the extent of rancidity, freshness, and oxidative stability of butter. Peroxides are intermediate products that break down into secondary oxidation products like aldehydes or ketones during the oxidative deterioration process, and these can be detected by the Kreis reaction (Kreis R.). PV in butter indicates incipient oxidation stage, and Kreis R. points to an advanced oxidation state [49,50].

#### 2.3.20. Gravimetric Dynamics of the Butter Package System During Long-Term Storage

Identically portioned butter samples were wrapped in the films produced, and the films were thermally welded to their ends. Twelve samples were prepared for each film formulation, and for each interval, 3 samples/film formulation were analyzed. The packaged butter was compared with commercially wrapped and unpackaged butter. The samples thus obtained were immediately weighed with analytical precision for determining the initial system mass (M_1_). The samples were stored in refrigeration conditions (4 °C). Subsequent weighing (M_2_) was performed at pre-determined intervals: 4, 14, 43 and 77 days. Three tests were performed for each type of packaging. The net mass change (ΔM) in grams was calculated as the absolute difference:

ΔM = *M*_1_ − *M*_2_ (9)

#### 2.3.21. Determination of Peroxide Index by Iodometric Method and Kreis Reaction

The degree of primary oxidation of the lipid phase in butter was assessed by determining the peroxide value (PV) using an iodometric titration procedure adapted from the method described from Alshehri et al. [13]. Briefly, 5.0 g of packaged butter was accurately weighed (±0.001 g) into an Erlenmeyer flask, and 10 mL of chloroform was added. The mixture was shaken until the sample was completely dissolved. Subsequently, 15 mL of acetic acid and 1 mL of freshly prepared saturated aqueous potassium iodide (KI) solution were added. After homogenization, the mixture was kept in the dark for 5 min to allow for the release of molecular iodine (I_2_) proportional to the amount of lipid hydroperoxides present. The reaction was stopped by adding 75 mL of distilled water, and the liberated iodine was titrated with 0.1 N sodium thiosulfate solution using freshly prepared starch solution (1 g/100 mL) as the indicator. A reagent blank was prepared under identical conditions, without the addition of butter.

The method was adapted to allow for the direct analysis of butter without prior separation of the fat phase. Considering the fat content of butter (82%), the 5.0 g sample corresponded to approximately 4.10 g of fat. This value was used for the calculation of the peroxide value on a fat basis.

The peroxide value was calculated as follows:

(10) $$\mathrm{PV}\;=\;\frac{\left(V_{1}-V_{2}\right)\cdot n}{m}\;\cdot 1000\;\mathrm{milliequivalents}/\mathrm{kg}$$

where V_1_—volume sodium thiosulphate solution used in the sample titration (mL).

V_2_—volume of sodium thiosulphate solution used in the control sample titration (mL).

m—mass of the sample to be analyzed taken for determination (g).

n—normality of sodium thiosulphate solution used for titration (0.1).

The Kreis R. was carried out by determination of fat oxidation. By means of this reaction, the presence of epoxy aldehydes resulting from the oxidative processes of linoleic acid at an advanced stage of fat oxidation is assessed. At this stage organoleptic changes in taste, smell and color occur. In an acidic environment (HCl), the epoxy aldehydes react with phloroglucinol to form a colored compound. The intensity of the color depends on the amount of epoxy aldehydes, so proportional to the intensity of the oxidation process.

About 1 g of melted sample at about 50 °C was inserted in a test tube, followed by the addition of the same volume of HCl solution (37%) and of the same volume of 1% etheric solution of phloroglucinol. The test tube was shaken several times and then left in a stand at rest, watching for the appearance of a color from pink to intense red, depending on the amount of epoxy aldehydes present in the sample.

Interpretation of the Kreis reaction:

Negative: the content of the test tube remains colorless or has a slightly yellowish tinge.

Positive: the content of the test tube is colored red at different intensities, depending on the degree of rancidity.

### 2.4. Statistical Analysis

All experiments were performed in triplicate, with the mean and standard deviation calculated. The statistical significance was determined in Microsoft Excel, using ANOVA ONE-WAY processing, the TUKEY model.

## 3. Results and Discussion

### 3.1. Characterization of Films and Their Extract

**Figure 1:**
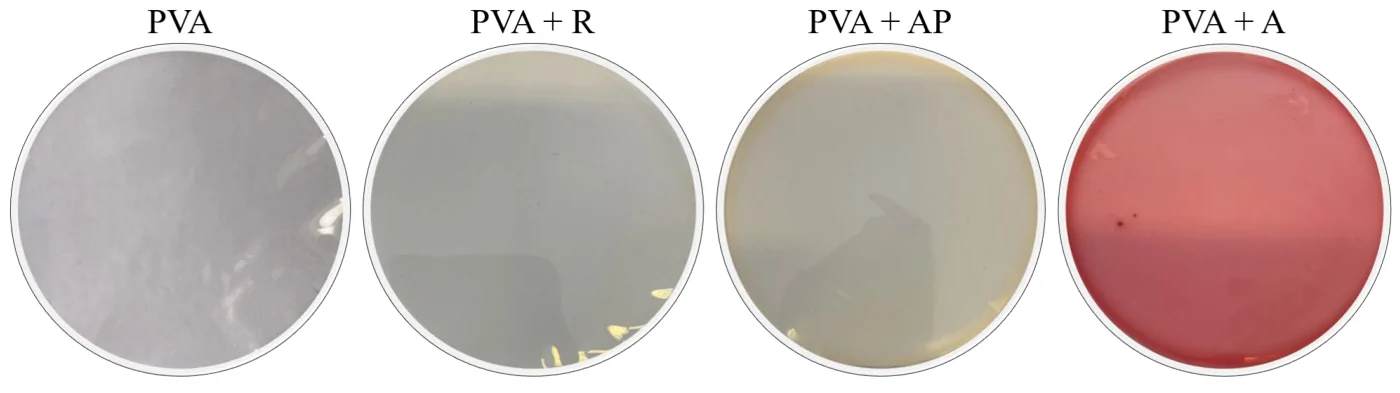
Macroscopic image of the films obtained, where R—rutin; AP—apple peel; A—aronia.

The PVA-based film was prepared and modified with natural extracts of aronia, apple peel, and rutin to obtain a potential packaging material with functional properties to be used for the packaging of foods with high oxidation potential. The macroscopic image of the film obtained can be observed in Figure 1.

The PVA film was homogeneous, transparent, flexible, colorless, 0.11 ± 0.01 mm thick, and was used as a reference film to be compared to the modified films. The PVA + R film had a translucent, homogeneous, flexible appearance with a very slight tinge of yellow and had a thickness of 0.10 ± 0.01 mm. The PVA + AP film was homogeneous, transparent, with a slight tinge of yellow to pink and had a thickness of 0.13 ± 0.02 mm. The PVA + A film was homogeneous, less transparent than the other films obtained, with a reddish tint and had a thickness of 0.11 ± 0.01 mm. Similar results were reported by Roy and Rhim [15], which indicated that adding rutin to the chitosan gelatin binary composite films changed the color of the reference film to a bright yellow. Furthermore, Riaz et al. [28] demonstrated that by adding apple peel polyphenols (APPs) to the chitosan, the film acquired a reddish color, which became more pronounced as the APP concentration increased in the film. The authors conclude that this may be beneficial for protecting food against UV-Vis light by preventing nutrient loss, discoloration and the formation of unpleasant odors. Also, Long et al. [23] showed that the color of chitosan/PVA films with *Aronia melanocarpa* extract ranged from red to pale pink, purple/blue and yellow, depending on the pH of the buffer solution, due to the chemical changes that take place in the anthocyanins structure. Anthocyanins, at a low pH, give a red color to the extract due to their cation form flavilium.

### 3.2. Determination of Polyphenols Compounds by Reversed-Phase High-Performance Liquid Chromatography with Photodiode Array Detection (HPLC-PDA)

**Figure 2:**
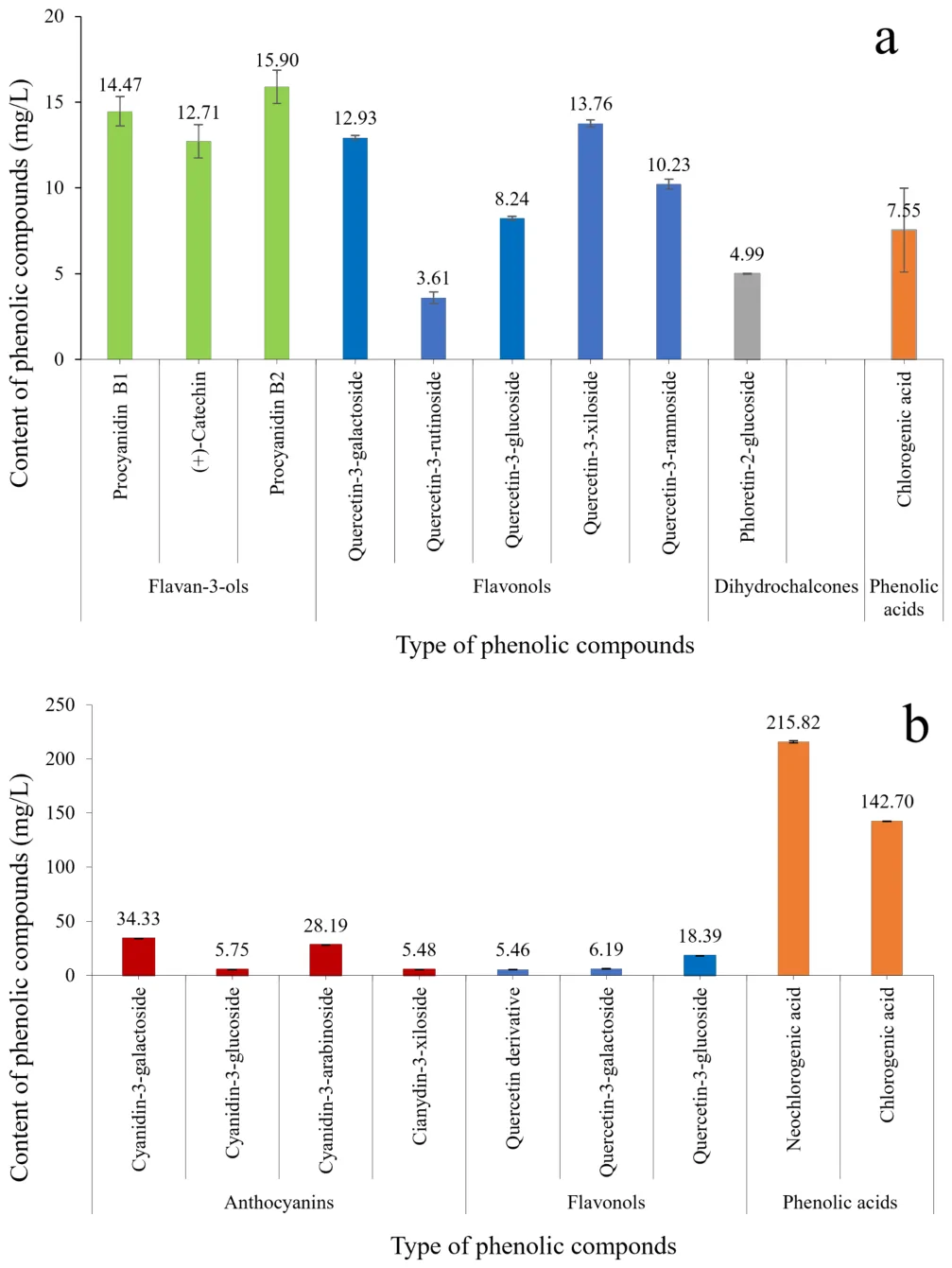
The content of individual polyphenols in apple peel extract (**a**) and aronia extract (**b**).

HPLC analysis was performed to study the content of phenolic compounds in the extracts obtained. The individual polyphenols identified in the apple peel extract can be seen in Figure 2a and those in the aronia extract in Figure 2b.

Four classes of polyphenols were identified in the apple peel extract, namely flavan-3-ol, with values ranging from 12.71 ± 0.97 to 15.90 ± 0.96 mg/L; flavonols, with values ranging from 3.61 ± 0.33 to 13.76 ± 0.2 mg/L; from the class of phenolic acids, chlorogenic acids with 7.55 ± 0.2 mg/L; and from the dihydrochalcones class, phloretin-2-glucoside with 4.99 ± 0.03 mg/L was identified. Similar results were obtained by Ferreira et al. [26], who studied the phenolic composition of Golden and Red Delicious apples.

Polyphenols from aronia extract belong to three different subclasses, namely anthocyanins, which were present in quantities of 5.48 ± 0.02 to 34.33 ± 0.03 mg/L; flavonols, with values ranging from 5.46 ± 0.004 to 18.39 ± 0.004 mg/L; and phenolic acids, in the amount of 142.7 ± 0.003 to 215.82 ± 0.94 mg/L. Similar results were reported by Talebi et al. [51], which characterized the anthocyanins profile of powdered Aronia extract incorporated with chitosan/kappa-carrageenan-based edible films with the addition of ginger oil nano-emulsion.

### 3.3. Morphology of the Films Obtained and Elemental Analysis

**Figure 3:**
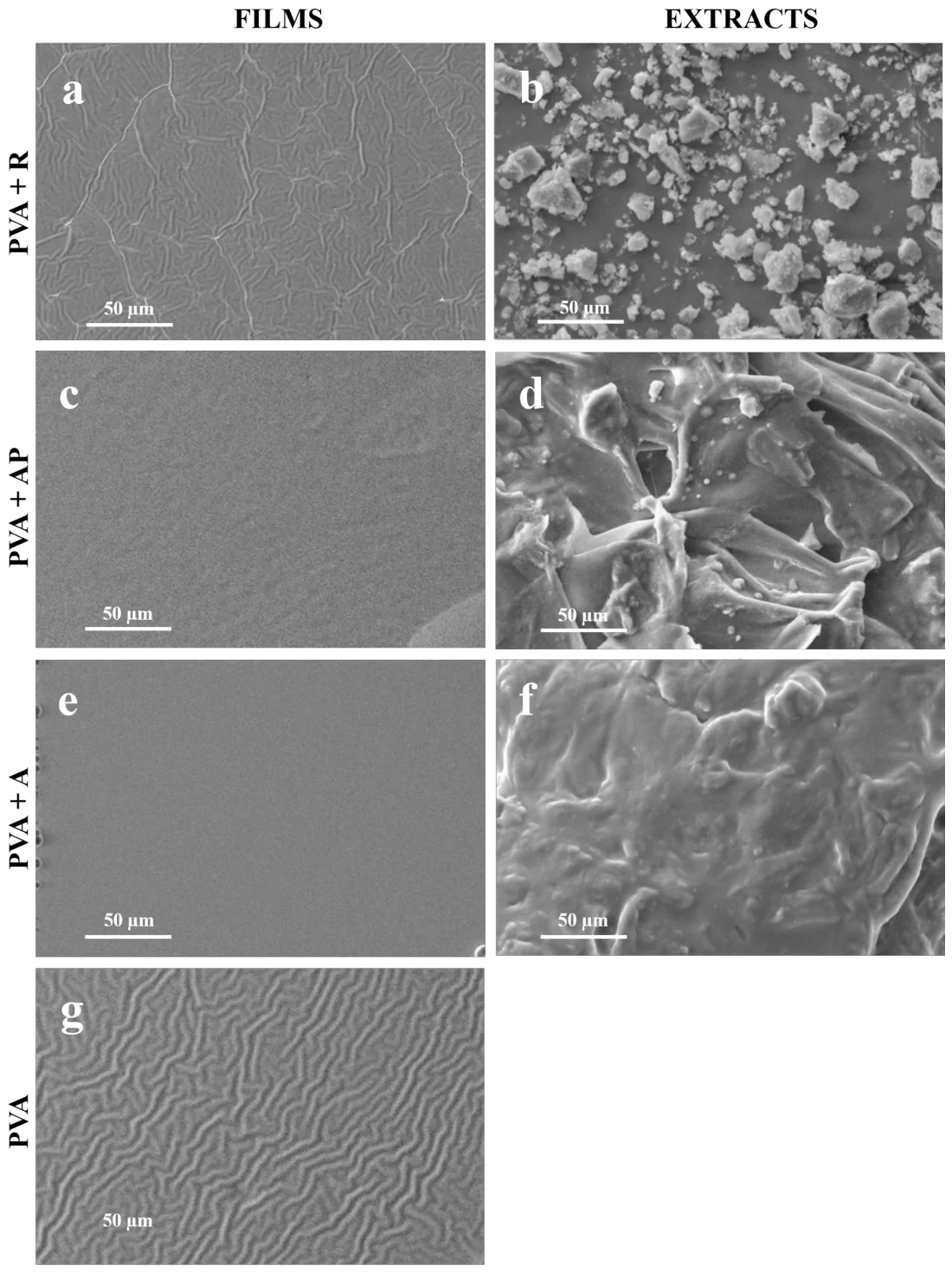
SEM images of the surface of the extracts used and the films obtained at 50 µm for PVA + R (**a**,**b**), PVA + AP (**c**,**d**), PVA + A (**e**,**f**) and PVA (**g**). Where R—rutin; AP—apple peel; A—aronia.

The SEM analysis was carried out to examine how polyphenolic extracts affected the reference film’s microstructure, providing information on the surface morphology of films. The SEM images can be observed in Figure 3.

The SEM micrograph of the reference film revealed a homogeneous and compact surface characterized by pleated and interspersed chains, without the presence of cracks or gaps, likely due to the film-forming properties of PVA, as Van Nguyen and Lee [29] pointed out.

The microstructure of the PVA + R film was more homogeneous than that of the reference film. It was compact, without cracks or gaps, but with small conglomerates, as can be seen in the microstructure of the rutin. The microstructure of the PVA film was improved by adding rutin. The results obtained are consistent with those of Narasagoudr et al. [33], who demonstrated that the surface morphology of the chitosan film and PVA was improved by adding rutin, due to the compatibility of rutin with the other components of the matrix.

Even though the surface morphology of the AP extract showed a rough surface, the micrograph of the film’s surface PVA + AP was smooth, compact and without striations or cracks, which indicates good miscibility between the film components. These results are consistent with the FTIR and XRD analysis, indicating the compatibility and interaction between the components of the PVA + AP matrix by the presence of a semicrystalline structure. Similar results were reported by Riaz et al. [28], who added apple peel extract to the chitosan film and obtained surface micrographs of smooth and even blending films, even at lower extract concentrations. The results are also confirmed by Van Nguyen and Lee [29], who showed that the addition of apple peel extract (APE) resulted in a more uniform dispersion of the PVA-based nanocomposite, cellulose nanocrystals and titanium dioxide, due to the high interaction of APE with the film components by hydrogen bonds.

Aronia demonstrated its compatibility with PVA film by forming a very compact, smooth and crack-free network, probably due to the strong intermolecular hydrogen bonds created by introducing aronia extract, resulting in a good dispersion and distribution in the PVA matrix. The results of FTIR spectra and XRD analysis also confirm the compatibility of aronia extract with PVA film, indicating the ability of the Aronia to change the molecular order of PVA film. Similar results were indicated in Long et al. [23] by adding aronia extract to the film matrix of PVA and chitosan; due to the presence of anthocyanins and phenolic acids in its composition, they obtained a smoother and wrinkle-free surface. Furthermore, Wu et al. [24] prepared starch and PVA films with the addition of aronia and indicated that the compatibility between the components of the film by forming new hydrogen bonds is improved when adding an adequate amount of aronia.

**Figure 4:**
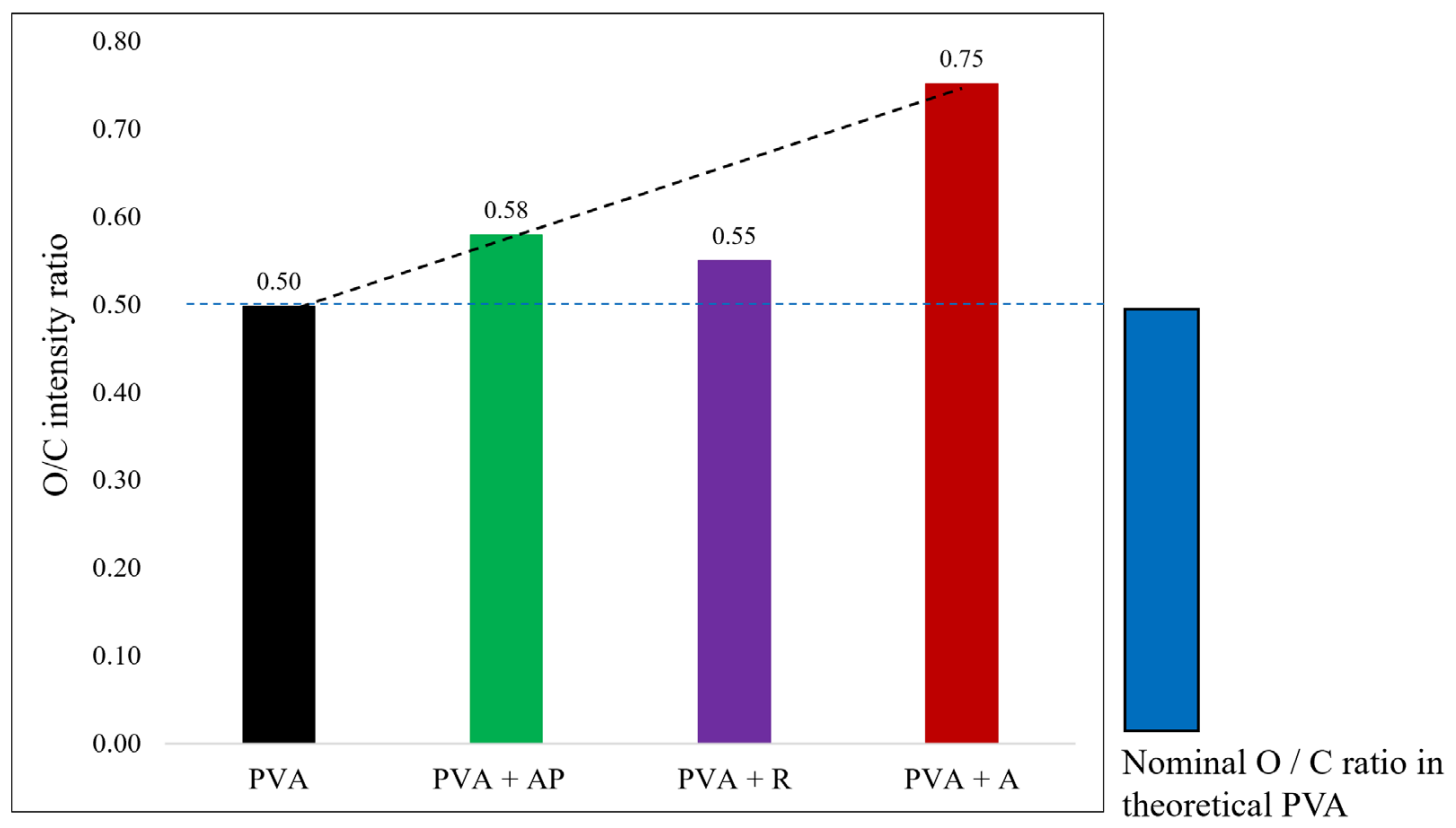
Oxygen-to-carbon (O/C) atomic ratio derived from EDXS analysis of the investigated samples, where R—rutin; AP—apple peel; A—aronia.

EDXS analysis was used to evaluate the elemental composition of the samples, and the oxygen-to-carbon (O/C) ratio was subsequently calculated. Carbon was present in the highest amount, at a percentage of about ~60%, followed by oxygen at a percentage of about 40% in PVA films. Very small amounts of potassium, calcium, magnesium, phosphor, and chlorine were present in the other films, in percentages less than 1%. The amount of carbon (compared to oxygen) decreases with the addition of polyphenols, as is apparent from the O/C ratio presented in Figure 4, which indicates the presence of polyphenols in the PVA matrix [52]. Increasing the O/C ratio could lead to increased hydrophilicity and antioxidant activity, as well as lower thermal stability.

**Figure 5:**
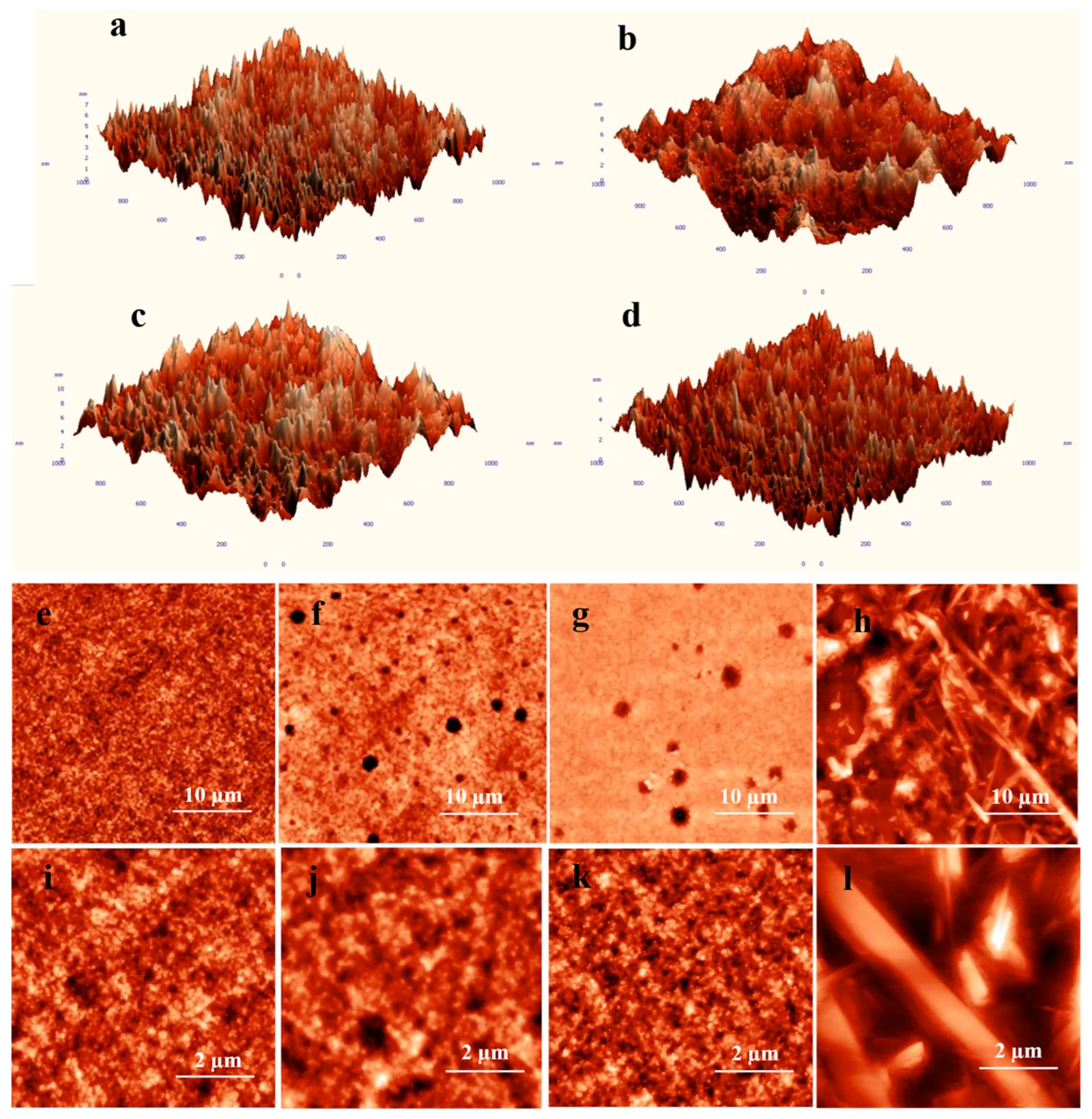
AFM height images in 3D and 2D depicting the surfaces of thin films of PVA (**a**,**e**,**i**), PVA + A (**b**,**f**,**j**), PVA + AP (**c**,**g**,**k**) and PVA + R (**d**,**h**,**l**), where A—aronia; AP—apple peel; R—rutin. Size of 3D images is 1 × 1 µm^2^ (**a**–**d**) and 2D is 2 × 2 µm^2^ (**i**–**l**) and 10 × 10 µm^2^ (**e**–**h**).

The three-dimensional maps obtained from AFM analysis showed relatively small morphological differences between the reference film and the three modified films, namely the structures formed by the reference film appear smaller in the AFM images of greater magnificence (1 × 1 microns), as can be seen in Figure 5a–d. Larger amorphous structures (dark color) were also observed in smaller magnification images (10 × 10 microns) of modified films, as shown in Figure 5e–h. Such amorphous structures were missing in the reference film. Small point structures (very light in color) that are not present in the reference film (Figure 5i) appear in PVA + AP films, as can be seen in Figure 5k. Such results have also been reported by Van Nguyen and Lee [29] who identified the presence of white spots on the surface of the PVA nanocomposite, cellulose nanocrystals, titanium dioxide and apple peel extract, attributed to polyphenolic compounds and sugars. Between the PVA + R and the reference film, there are visible differences, in the sense that the modified film shows crystalline/crystal structures (Figure 5l).

The mean and square roughness as well as the particle size were determined on topographic images of 1 × 1 microns and are shown in Table 1. The PVA film had the lowest mean and square roughness and smaller particle size, which indicates that it shows a smooth and homogeneous surface.

The surface topography of the film of PVA + R is like that of the reference film in terms of medium and square roughness, as well as particle sizes. This means that the rutin had a uniform dispersion in the film matrix, as evidenced by SEM analyses. Similar results were obtained by Koshy et al. [53], who added fillers, namely carbon points from protein soy isolates, *Boswellia serrata*, anthocyanin *Clitoraternatea* and palmira root, to the PVA film matrix and indicated that the modified films showed lower roughness than the reference film due to the uniform distribution of fillers.

The PVA + AP film showed greater roughness than the PVA + A film, while the particle size of the PVA + A film was larger than the PVA + AP film, both films having a roughness and particle size larger than the PVA film, but with very small differences. Similar results were obtained by Talib et al. [54], who prepared cadmium sulfide (CdS) and PVA nanocomposite films with the addition of thiourea in various mass proportions and studied the effect of thiourea on the polymer network. The AFM results indicated that the average roughness and the average square roughness of the surface of the nanocomposites obtained increased as the concentration of thiourea went up, the authors assuming that this would be due to the increase in particle sizes.

### 3.4. Structural Analysis of the Films Obtained

The FTIR analysis of the films obtained was performed to understand how the chemical structure of the PVA film was changed by possible interactions between the molecules of the film components. The FTIR spectra of the modified films were similar those of the reference film.

In the PVA spectrum, the broadband present at about 3270 cm^−1^ was attributed to -OH symmetrical stretching vibrations [14]. In the FTIR spectra of modified films, this band exhibits increased broadening and is shifted to approximately 3280 cm^−1^, indicating possible interactions between the hydrophilic groups of PVA and the highly reactive species -OH from the polyphenols’ composition willing to give up electrons [33].

Bond bending vibrations -OH and C-H scissoring, in flat deformation, attributed to alcohols and phenols, were found in the PVA spectrum at 1420 cm^−1^, and in the modified films spectra at lower wavelengths (i.e., 1416 cm^−1^ for PVA + AP and PVA + A, 1419 cm^−1^ for PVA + R); this shift in peaks suggests intermolecular interactions between the matrix components [23].

The bands corresponding to CH_2_ asymmetric stretching were present at 2923 cm^−1^ in the PVA spectrum, which shifted to 2926 cm^−1^ in the modified films spectra. Also, at 1324 cm^−1^ bending vibrations, out of plane, were recorded in the PVA spectrum, which corresponded to stretching vibrations of the C-O aromatic bands in the modified films. This signal shifted to 1332 cm^−1^ for PVA + AP. At 1266 cm^−1^ the CH_2_ stretching vibrations in the PVA spectra were recorded, these signals being attributed to C-O-C vibrations of aromatic groups in the modified films, which shifted to 1275 cm^−1^ for PVA + AP. Symmetric stretching vibrations of the C-O bonds were present at 915 cm^−1^ in all the FTIR spectra [55,56]. At 837 cm^−1^ there were also recorded rocking vibrations in the PVA spectrum, shifted to 839 cm^−1^ for PVA + A and PVA + R, and to 822 cm^−1^ for PVA + AP, these being anthocyanin-specific bending vibrations, out of plane, their displacement suggesting the binding of anthocyanins of PVA [29,57].

The bands observed at 1715 cm^−1^ in the PVA spectrum corresponding to the stretching vibrations of the C=O bond are attributed to the presence of CA in the PVA structure and are specific to the aromatic groups in the modified films, which are present at lower wavelengths. The displacement of the bands and the decrease in the signal intensity of the phenolic compounds indicate the possible interactions that occurred between matrix components [58,59]. The vibration of characteristic C=C bands of the aromatic rings were observed around 1650 cm^−1^ in the spectra of the PVA + A and PVA + AP films and around 1640 cm^−1^ for the PVA + R spectra, confirming the physical presence and encapsulation of extracts in the PVA matrix [12].

C-O stretching vibrations were present in the PVA spectrum at 1083 cm^−1^. These bands shifted at about 1039 cm^−1^ in the modified films, being also assigned to the aromatic C-H and presented the peaks of much higher intensity, this indicating potential interactions between the matrix components [33].

**Figure 6:**
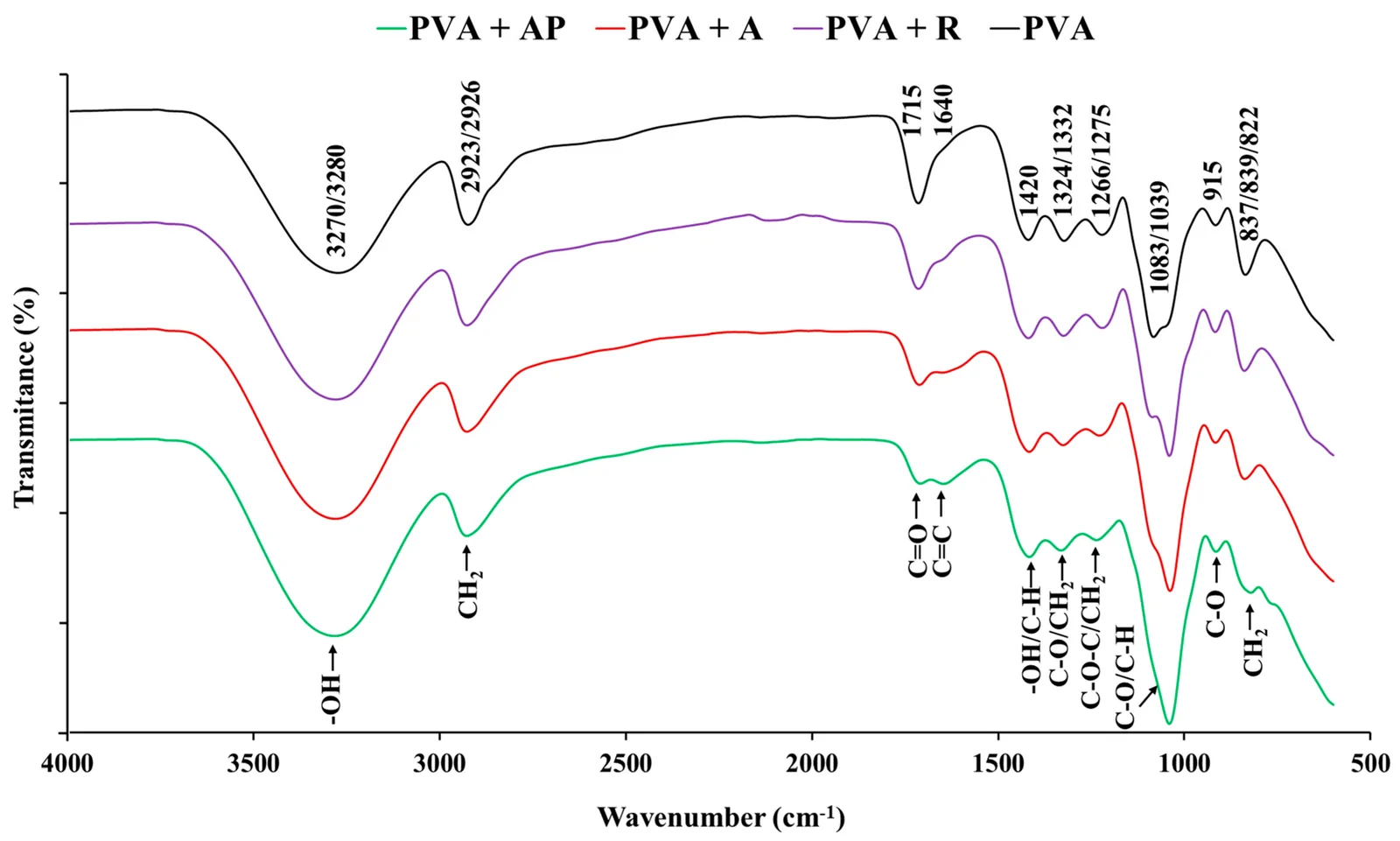
FTIR spectra for PVA, PVA + AP, PVA + A and PVA + R films, where A—aronia; AP—apple peel; R—rutin.

The displacement of peaks to a lower or higher wavenumber and the increase or decrease in peak intensity suggests changing the physical environment and structural reorganization by the possible interactions that occurred mainly between the hydroxyl groups from PVA network and the phenolic hydroxyl groups from phenolic acids, anthocyanins, flavonols, catechins and procyanidins present in the extracts. The results are confirmed by Oun et al. [13], who prepared composite films based on PVA and chitosan (CH) with the addition of aronia, grapefruit seed extract and cellulose nanocrystals and indicated that the displacement and the change in peak intensity would be due to the formation of hydrogen bonds between the PVA/CH matrix and the added materials. The FTIR spectra of the films produced can be seen in Figure 6.

**Figure 7:**
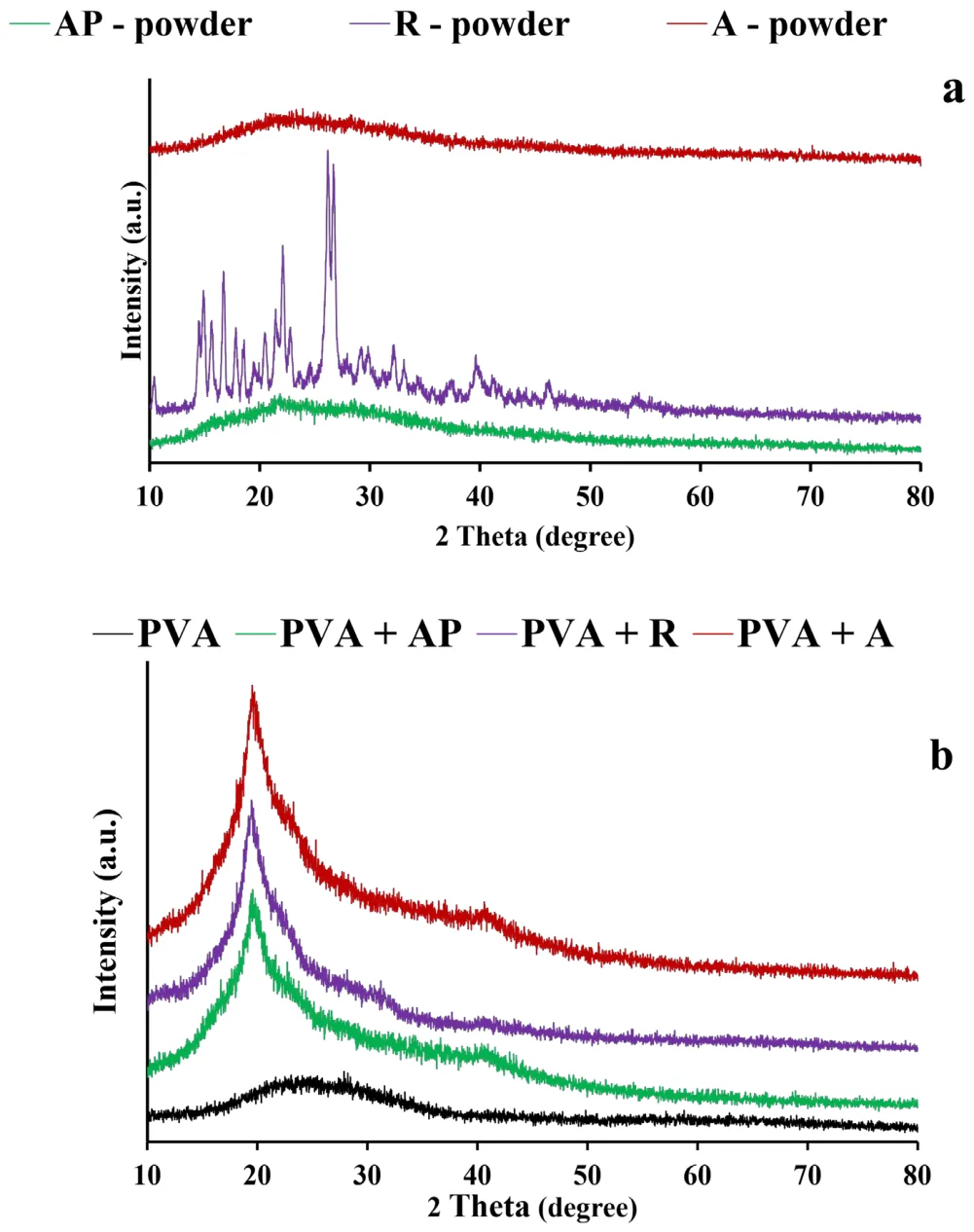
XRD diffraction profiles of extracts (**a**) and films obtained (**b**), where A—aronia, AP—apple peel and R—rutin.

The XRD structural analysis was conducted to study the effect of polyphenolic extracts on the crystallinity of the PVA film and to indicate the intermolecular interactions that are created between the components of bioactive films and the miscibility between them. Each polymer has its characteristic crystalline region. If there are interactions between two polymers, the molecular order can be altered, and if there are significant structural changes in the crystalline or intercalated phase, they can be observed in XRD patterns through variations in amorphous halo or diffraction peaks. The XRD diffraction profiles of the extracts and films obtained can be seen in Figure 7, and the relative degree of crystallinity (CI, %) can be seen in Table 2.

The PVA film displays a mostly amorphous structure, with a broad and low intensity halo, with some short-range molecular ordering, and with characteristic diffractions at 2θ = 19.7°, at 22.84° and at 28.22°, as indicated in Song et al. [59]. The PVA film does not show obvious crystalline diffraction, probably due to the presence of citric acid as a cross-linking agent in the film matrix that caused hydrogen bonds to form, preventing PVA crystallization, as indicated Jiang et al. [60], who prepared composite films of chitosan, cross-linked with citric acid and PVA, and indicated that the presence of citric acid and PVA in the chitosan matrix prevented the crystallization of chains by the formation of strong hydrogen bonds between the matrix components.

The apple peel extract presented an amorphous structure. The addition of apple extract to the PVA matrix initiated the crystallization process and a partially ordered arrangement of the polymer network was promoted. The PVA + AP film shows a CI by 17.21 ± 0.39% with a maximum diffraction 2θ = 19.58°, perhaps due to the possible intermolecular interaction between the apple extract and the PVA matrix through hydrogen bonds between hydroxyl groups of PVA and phenolic acids (such as chlorogenic acids) found in the apple extract. These results are also supported by the FTIR and SEM analysis of the PVA + AP film, which showed a compact surface and indicated the compatibility between the matrix components. These results were also confirmed by Liu et al. [1], who prepared hydrogel films from PVA, apple polyphenols (APP), and polyacrylamide (PAM) and indicated a higher crystallinity of PVA/APP and of the PVA/PAM/APP films as compared to pure PVA film.

The XRD pattern of the rutin showed a partial crystalline structure with multiple diffractions at 2θ = 14.88°, at 16.7°, at 22.08°, at 26.18°, and at 26.68°. The PVA + R film registered CI of 18.83 ± 0.24% with a maximum reflection characteristic of the structure of PVA at 2θ = 19.46°, which suggests the interaction of rutin with PVA by hydrogen bonds and miscibility between the components of the matrix. The rutin has proven its ability to partially change the crystallinity of PVA film by changing the orientation and horizontal arrangement of PVA chains. The results were also confirmed by Narasagoudr et al. [33], who obtained materials based on chitosan and PVA with rutin in various mass proportions and found that the degree of crystallinity of the matrix increases as the content of rutin also increases.

The XRD pattern of the aronia extract showed an amorphous nature. The addition of aronia extract to the PVA film affected the original arrangement of the PVA film probable by forming intermolecular hydrogen bonds inside the matrix. The film PVA + A registered a CI of 19.92 ± 0.48%, with a characteristic PVA reflection at 2θ = 19.54°, which suggests that aronia had the ability to enhance the growth of PVA crystals and induced a high degree of miscibility between the components of the matrix. Initiation of the crystallization process was also supported by the FTIR and SEM analyses of the PVA + A film that revealed a uniform surface morphology, which suggests the compatibility of aronia extract with the PVA matrix and the possible intermolecular interactions that took place between the film components. Comparable findings were reported by Hosseini et al. [61], who produced chitosan and PVA mats loaded with peptides through electrofiling. Their results suggested that the alteration in film crystallinity is attributed to the strong intermolecular interactions among the matrix components, which reduce the intramolecular bonds of PVA. Without these interactions, the XRD patterns would merely reflect simple mixtures of the matrix components. Also, Song et al. [59] indicated that oligomeric proanthocyanidin nanospheres (OPCMs) introduced into the PVA matrix were able to increase the crystallinity of the film by inducing the horizontal alignment of the PVA chains.

The addition of polyphenolic compounds to the PVA matrix led to the alteration of the molecular order and the initiation of the crystallization process.

**Figure 8:**
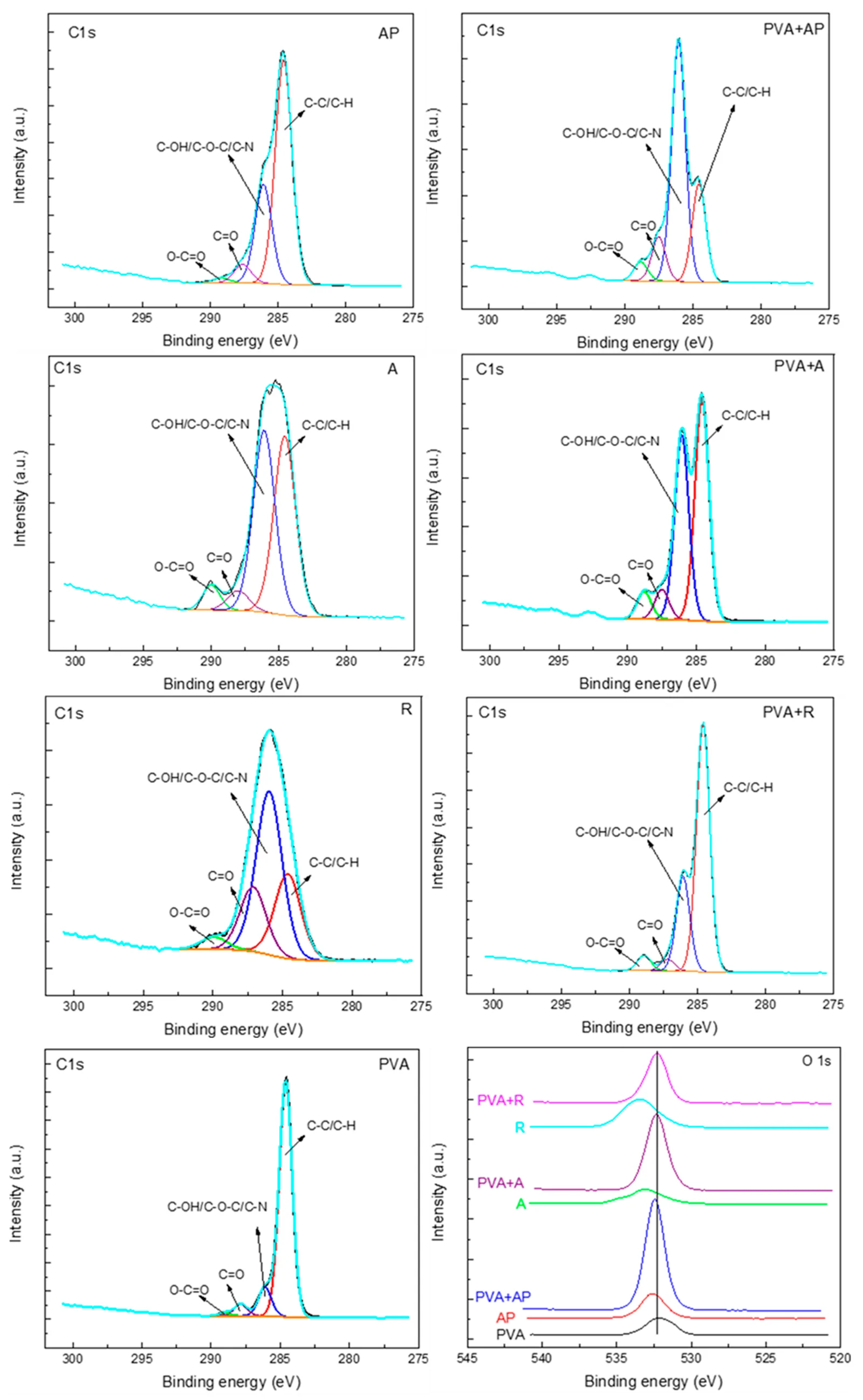
Deconvolution of C1s and O1s XPS spectra of extracts and film obtained, where A—aronia; AP—apple peel; R—rutin.

The XPS analysis was conducted for a deeper understanding of the interaction of polyphenols with PVA matrix and the presence of polyphenols on the surface. The high-resolution XPS spectra of C1s and O1s were collected and can be seen in Figure 8. The deconvoluted data of the C1s indicated four types of carbon species around 284.6, 286.1, 287.9, and 289 eV, which could be attributed to C-C/C-H, C-OH/C-O-C/C-N, C=O and O-C=O group, respectively. The related areas of C-C/C-H peaks were highest for the PVA, as the carbon skeleton of PVA is predominantly composed of the C-C single bonds and the functional groups -OH were present in a small share [62]. By adding polyphenols, the peak intensities for C-OH/C-O-C/C-N of the modified films were significantly enhanced compared to reference film, due to the intake of etheric and hydroxyl phenolic bonds. The binding energy (B.E.) of C=O shifted from 287.9 for PVA to 287.5 for PVA + AP and PVA + A with enhanced signal intensity, and to 287.2 for PVA + R with decreased signal intensity. The binding energy of O-C=O shifted from 289 for PVA film to 288.8 for PVA + AP and PVA + A, and then to 288.9 for PVA + R with the intensified signal. This spectral behavior indicates the changing of the chemical environment and potential interactions between the hydroxyl group in PVA, and the carbonyl and carboxyl groups present in the polyphenol structure [63].

The deconvolved O1s spectra contained a peak assigned to C-O/-OH, with the binding energy at 532.4 eV for PVA, PVA + A and PVA + R, while the binding energy for PVA + AP shifted at 532.5 eV. The related areas of oxygen in bound forms (phenolic OH) in the modified films also were significantly enhanced compared to PVA. Comparing the XPS spectra of PVA film to PVA-based films modified with phenolic compounds, the intensification of C-O, C=O, O-C=O signals from the C1s and O1s spectrum indicated the presence of polyphenols on the surface, which may be very significant for the antioxidant and antimicrobial activity of films. The change in local chemical environment was evidenced by the shifted in the position of the peaks C=O and O-C=O, as well as the changes in the intensity ratio between C-C and C-O. This suggests physical interfacial interactions between PVA and the functional groups of phenolic compounds (e.g., phenolic hydroxyl, carboxylic and carbonylic acids, sugars, etc.), indicating to the compatibility of PVA-polyphenol matrices [16,63].

These findings correlate with the increase in the O/C ratio observed in the SEM analysis, the broadening of the -OH band in the FTIR spectra, and structural modifications in the crystalline or intercalated phases of the modified films, as revealed by XRD and AFM analysis.

Based on the analysis of morphological and structural data, it can be concluded that incorporating polyphenols into the PVA matrix promotes intermolecular and interfacial interactions through hydrogen bonds as determined by the EDXS, FTIR and XPS analyses, as well as modification of molecular order indicated by XRD results. These intermolecular interactions could lead to a variation of physical–chemical proprieties of the films, such as barrier properties, antioxidant and antimicrobial capacity, as well as thermal stability, which will be discussed in the following sections.

### 3.5. Thermogravimetric Analysis and Differential Thermal Analysis (TGA/DTA)

**Figure 9:**
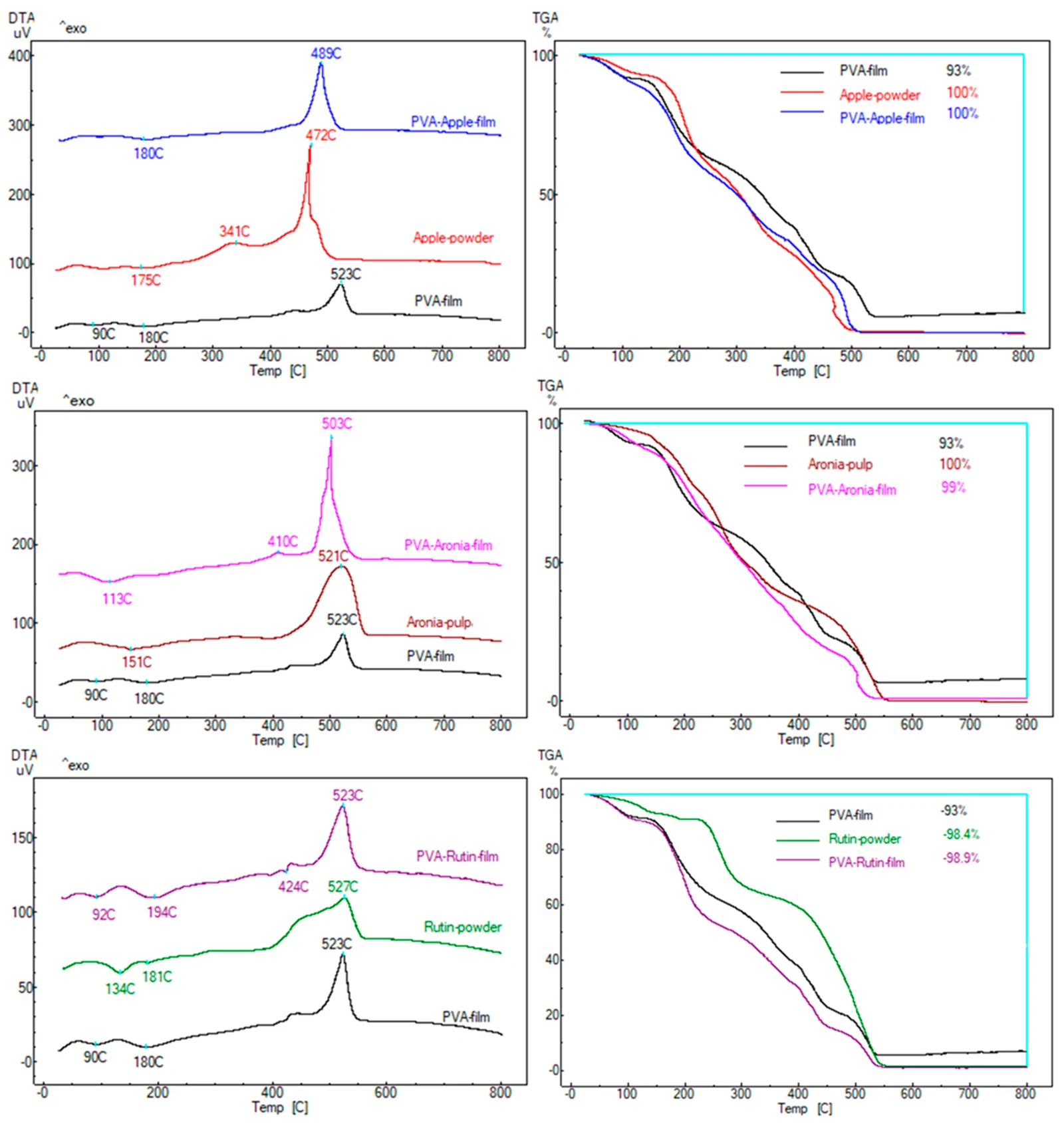
DTA/TGA curves of extracts and films obtained.

To investigate the effect of adding phenolic compounds on the thermal stability and degradation of PVA films, the samples investigated were analyzed by DTA/TGA. The DTA curves and TGA thermograms of the films obtained with rutin, apple and aronia extracts were compared with the DTA and TG curves of the PVA films and the extracts of the phenolic compounds used Figure 9. From the analysis of the DTA/TGA curves obtained for the analyzed samples, it is observed that during the heating of the samples, thermal events occur, accompanied by mass losses in several stages.

Thus, from the analysis of the DTA/TGA curve obtained on the PVA film without the addition of antioxidants, it is observed that in the 30–210 °C temperature range, two endothermic signals appear: the first, a peak at 90 °C (DTA curve) and a mass loss of ~8.5% (TG curve), is due to the loss of unbound water molecules, and the second endothermic signal, a maximum at 180 °C and a mass loss of ~21.3% (TG), is due to the vaporization of glycerol [64]. Between 210 and 385 °C, the cleavage of the polymer chain and degradation occur, with a mass loss of 30.2%. Between 385 and 550 °C, exothermic signals are evidenced with maxima at 443.6 and 523 °C, which corresponds to the breaking/degradation of the polymer backbone, with a mass loss in two stages, a total of ~34.4%, and a total residue of ~5.8% [65].

From the analysis of the DTA/TGA curves obtained on the samples with polyphenols, it is observed that the mass losses due to dehydration occur at temperatures close to that of the PVA film, but the degradation occurs at a lower temperature for PVA + AP (489 °C) and PVA + A (503 °C), compared to the PVA film (523 °C), except PVA + R (523 °C) whose degradation temperature coincides with that of the reference film.

From the comparison of the TGA curves of the samples improved with polyphenols with the TGA curve of the PVA film, it is observed that the total mass loss is between 98.9 and 100%. Thus, in the case of PVA + AP it is 100%, for PVA + R it is 98.9% and, respectively for PVA + A is 99%, compared to PVA where it was found to be 93%, which could be explained by the reduction of carbonic materials existing in the PVA matrix dissociated into volatile fragments [13].

Adding rutin did not change the thermal stability of PVA film, as both degraded at the same temperature. PVA + AP and PVA + A films showed lower degradation temperatures than PVA film, likely due to phenolic hydroxyls destabilizing the PVA network.

These results are in line with SEM, FTIR and XPS analyses that more dense hydrogen bond network. However, this may bring improvements in terms of polymer degradability. Similar results were obtained by Riaz et al. [28], who found that adding apple peel polyphenols decreased the thermal stability of chitosan film by hindering CS chain interactions through hydrogen bonds or free polyphenols. Similarly, Long et al. [23] indicated that by adding aronia in the PVA and chitosan film significantly reduced the degradation temperature of PVA due to the destruction of intramolecular hydrogen bonds.

### 3.6. Water Vapor Permeability (WVP)

WVP is one of the important characteristics of a packaging material, as the penetration of water vapors inside the packaging could influence the quality and safety of food, as well as its shelf life [66].

The film PVA + R had the lowest WVP values, measuring 4.11 ± 0.14 × 10^−10^ g/s·m·Pa. This was an improvement compared to the WVP of the PVA film, which recorded values of 4.26 ± 0.15 × 10^−10^ g/s·m·Pa. The PVA + A film recorded values of 4.50 ± 0.21 × 10^−10^ g/s·m·Pa, slightly higher than the reference film. The PVA + AP film exhibited higher WVP values compared to the reference film, specifically 5.36 ± 0.07 × 10^−10^ g/s·m·Pa. The ANOVA One-Way statistical analysis using the Tukey model showed no significant differences between PVA film and PVA modified with aronia extract or rutin. This indicates that the WVP of the reference film remained unaffected. The results are corroborated by XRD analyses, which reveal semicrystalline networks that are well cross-linked, featuring tortuous paths that are challenging to penetrate and demonstrating good crystal distribution. Moreover, SEM results indicate smooth and homogeneous surfaces, attributed to the robust intermolecular hydrogen bonds formed between the functional groups of PVA and phenolic groups. These results could be due to the hydroxyl groups in the structure of the aronia, which also serve as physical cross-linking [67] and a hydrophobic character of the rutin [32]. Narasagoudr et al. [33] indicated that the adding of rutin in chitosan and PVA films improved the water barrier properties due to the strong hydrogen bonds that were created between functional groups, as well as the compactness of the network and the homogeneity of the film surface.

Instead, significant statistical differences were observed between the PVA + AP film and the other films, indicating that the PVA + AP film did not enhance the WVP of the reference film. These results may be attributed to the voluminous structure of polyphenols, which insert between the long chains of PVA, reducing the intermolecular forces between them and increasing the free space (free volume) inside the polymer, water vapor molecules can diffuse more easily and quickly through film and, to higher average roughness, as indicated by the AFM analysis, which suggests weaker molecular interconnectivity, as documented in the literature [68,69,70]. Furthermore, the SEM-derived O/C ratios, together with the FTIR spectra, suggest a substantial presence of hydrogen bonds, which may enhance the film’s interaction with water and influence its overall hydrophilicity and degradability. Similar results were obtained by Van Nguyen et Lee [29], which indicated that the addition of the apple peel extract led to the increase of the WVP of the PVA/cellulose nanocomposite matrix and suggested that it was probably due to the hydrophilic character of the apple peel extract.

### 3.7. Changes of Permeability to Fat

Fat permeability is an important feature of a packaging material as it can indicate the ability of the material to prevent fat from passing through its structure. An adequate barrier against fats can prevent the spoilage of packaged products and the loss of food quality and safety [71]. The fat permeability of the film obtained has been tested against castor oil and all modified films prevented castor oil from passing through the film, both at 4 °C and at 20 °C. PVA-based films modified with phenolic compounds could represent potential packaging materials for high-fat foods.

### 3.8. Variation of the Permeability to UV-Vis Light

**Figure 10:**
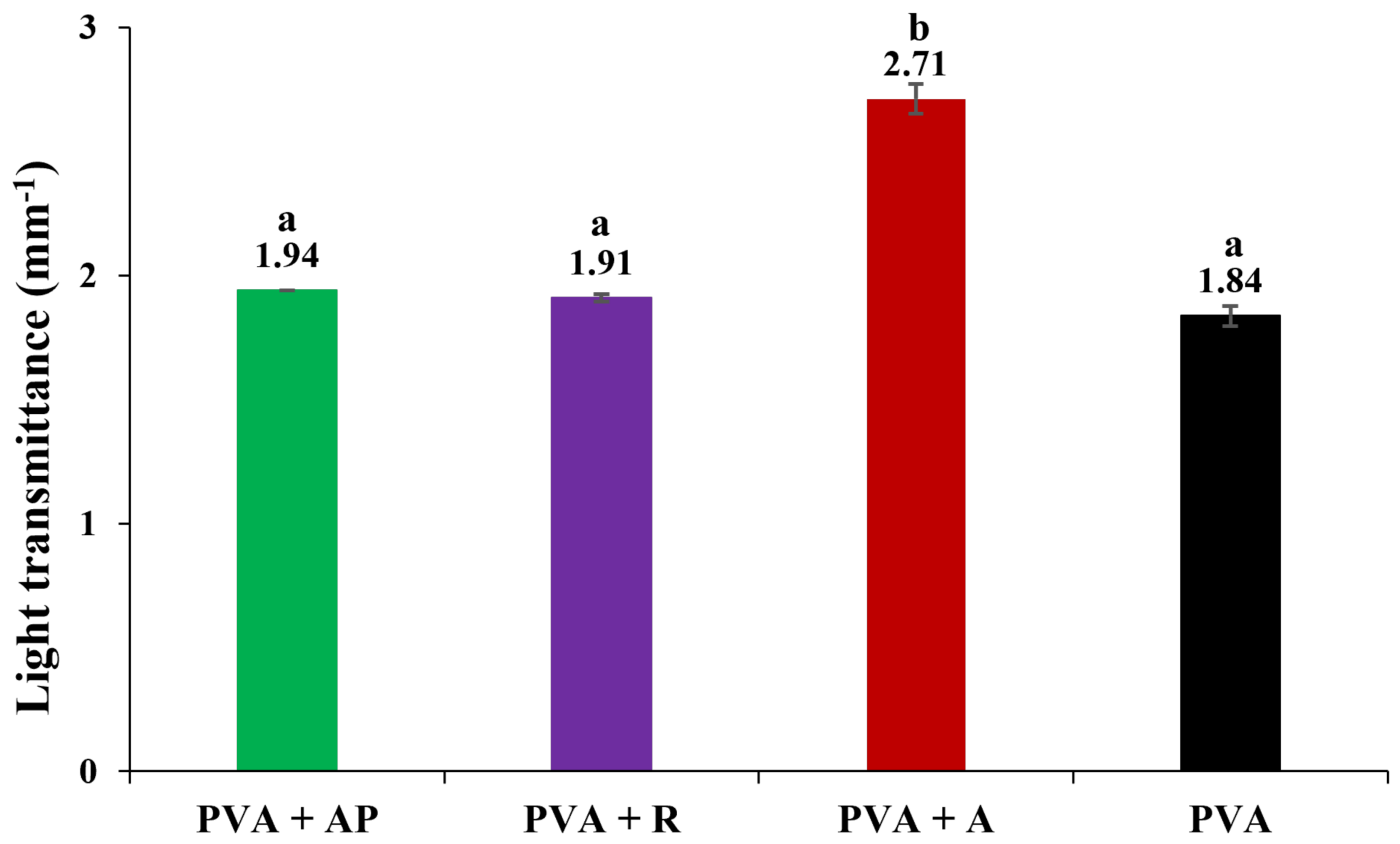
The light transmittance of the films obtained, where A—aronia; AP—apple peel; R—rutin. According to ANOVA One Way, using the Tukey model, when the same letter is used, there is no statistically significant difference between values. Different letters indicate that the difference between values is statistically significant at a level of more than 95%.

High barrier properties against UV-Vis light could ensure lower light transmission, which is desirable in a packaging material because it can prevent lipid oxidation and degradation of UV-Vis light-induced food [33]. The light transmittance of the film obtained can be observed in Figure 10.

The PVA + A film shows higher barrier properties against UV-Vis light than the reference film, probably due to the abundance of aromatic rings present in the structure of anthocyanins having UV-Vis radiation absorption property [72]. This film improved the barrier properties of the reference film against UV-Vis light with 47%, being statistically different. The PVA + A film could be a packaging material for foods that require protection against light-induced degradation, such as oily products. Similar results were obtained by Ma et al. [58] and Wu et al. [24], who indicated that with the incorporation of anthocyanins from aronia and mulberry in the films matrix the barrier properties against UV-Vis light increased, due to the change in the color of the films and the change in the chemical structure of the anthocyanins present in the extracts.

The PVA + R and PVA + AP films demonstrated light transmittance values close to those of the PVA film, with no statistically significant differences observed between them. Similar results were indicated by Van Nguyen et Lee [29], which concluded that the addition of apple peel extract to the PVA film and cellulose nanoparticles did not alter the barrier properties against UV-Vis light. Zhang and Zhao [73] indicated that barrier properties against UV-Vis of corn starch films modified with zein–rutin composite nanoparticles increased as the percentage of rutin added increased, greater protection against UV-Vis light providing films with higher content of rutin.

### 3.9. Water Absorption (Equilibrium Water Content—EWC) and Wetting

**Figure 11:**
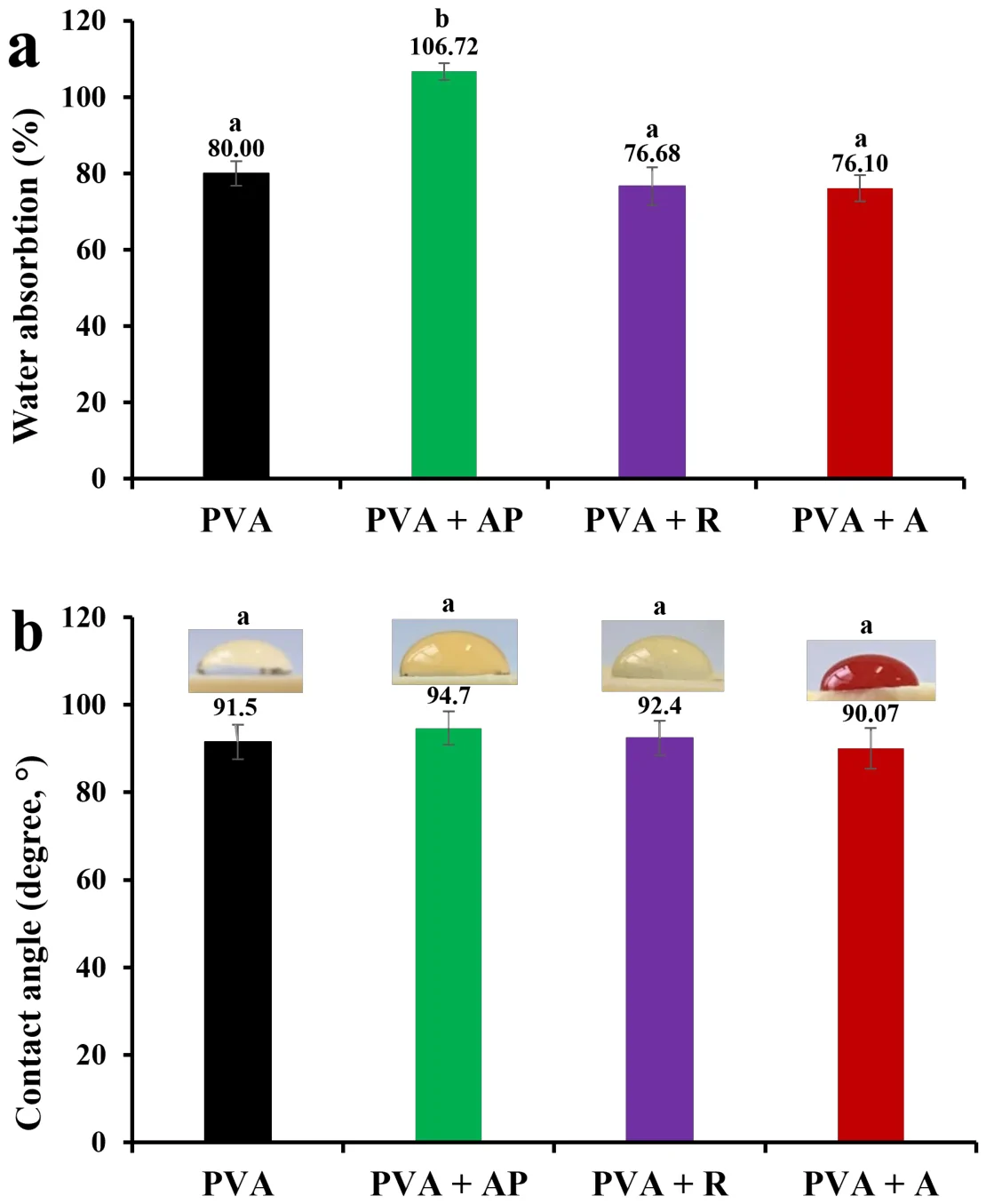
Absorption of water in the obtained films (**a**) and the values of the contact angle formed by dripping the film forming solution on the surface of a piece of butter (**b**), where A—aronia; AP—apple peel; R—rutin. According to ANOVA One Way, using the Tukey model, when the same letter is used, there is no statistically significant difference between values. Different letters indicate that the difference between values is statistically significant at a level of more than 95%.

Water absorption is an important feature of a packaging material as it can extend the shelf life of food safely by minimizing the risk of contamination or damage [74]. Figure 11a shows the percentage of water absorption of the films obtained.

Polyvinyl alcohol (PVA) exhibits hydrophilic properties due to the numerous hydroxyl groups along its polymer chain, which readily interact with water. Consequently, the PVA film demonstrated water absorption values of 80 ± 3.22%. The PVA + AP film showed the highest water absorption value at 106.72 ± 2.23%. This is likely due to the apple extract’s high hydrophilicity, phenolic hydroxyl groups, and the weakening of intermolecular interactions in the polymer chain, which promotes water absorption. Another reason could be the higher average roughness as indicated in the AFM analysis. These results align with the WVP analysis, this film showing the higher WVP. Similar results were obtained by Riaz et al. [28], who indicated that the addition of apple peel polyphenols to the chitosan matrix improved the film hydrophilicity due to the easy binding of phenolic hydroxyl groups with water molecules. The PVA + A film had water absorption values of 76.10 ± 2.5%, and the PVA + R film recorded 76.68 ± 4.96%, comparable to the values of PVA film. This could be due to the strong interactions that have been created between the highly reactive hydroxyl groups of phenolic compounds and PVA, leaving few hydroxyl groups available for water binding due to the competitive binding phenomenon, creating a sinuous path difficult to penetrate. These results are also supported by WVP analysis, which indicated WVP values comparable with the reference film. Furthermore, SEM and XRD analyses showed compact and homogeneous surfaces and more ordered networks harder to penetrate. Similar results have been obtained by Hasan et al. [74], who prepared films of purple sweet potato starch/chitosan nanoparticles with the addition of anthocyanin extract, indicated lower water absorption with the addition of anthocyanins due to the formation of a complex structure due to the interaction between the components of the matrix, limiting the mobility of the water molecule by reducing the free volume in the film.

Wetting is the ability of a liquid to maintain contact with a solid surface. This was assessed by determining the contact angle that was formed at the contact of the film-forming solution with a piece of butter, to obtain information about how the films obtained could protect high-fat food. The interaction of packaging with food can affect food preservation, the presence of food residues on the packaging surface can accelerate oxidation processes that lead to loss of quality and nutritional values of the food. A contact angle of less than 90° indicates wetting, and a contact angle greater than 90° non-wetting, and a contact angle of 90° may indicate either wetting or non-wetting [75]. As demonstrated in the literature, both PVA and phenolic compounds exhibit hydrophilic characteristics due to the presence of hydroxyl groups on the PVA chain and those on the aromatic rings of polyphenols [28]. The results obtained are presented in Figure 11b.

The contact angles of the drop formed by the film-forming solution and the piece of butter for the modified films were a little bigger than 90 °C, with no statistical differences from those of the PVA film, which would suggest that the films might not be wetting the piece of butter. This phenomenon could be due to the difference in character between butter (hydrophobic character) and the film-forming solution (hydrophilic character). The literature reports that greater surface hydrophobicity corresponds to higher water contact angles, as observed in lotus leaves, whose waxy coating promotes droplet mobility [75]. The highest contact angle values were observed for the PVA + AP film, with θ = 94.7°. The lowest contact angle values were obtained by PVA + A film with θ = 90.07°, followed by PVA film with θ = 91.5° and PVA + R film with θ = 92.4°. The incorporation of phenolic compounds did not affect the wettability properties of the PVA film and its hydrophilic character. These results were in line with those of water absorption and WVP analysis.

### 3.10. Variation of Solubility in Food Simulants

**Figure 12:**
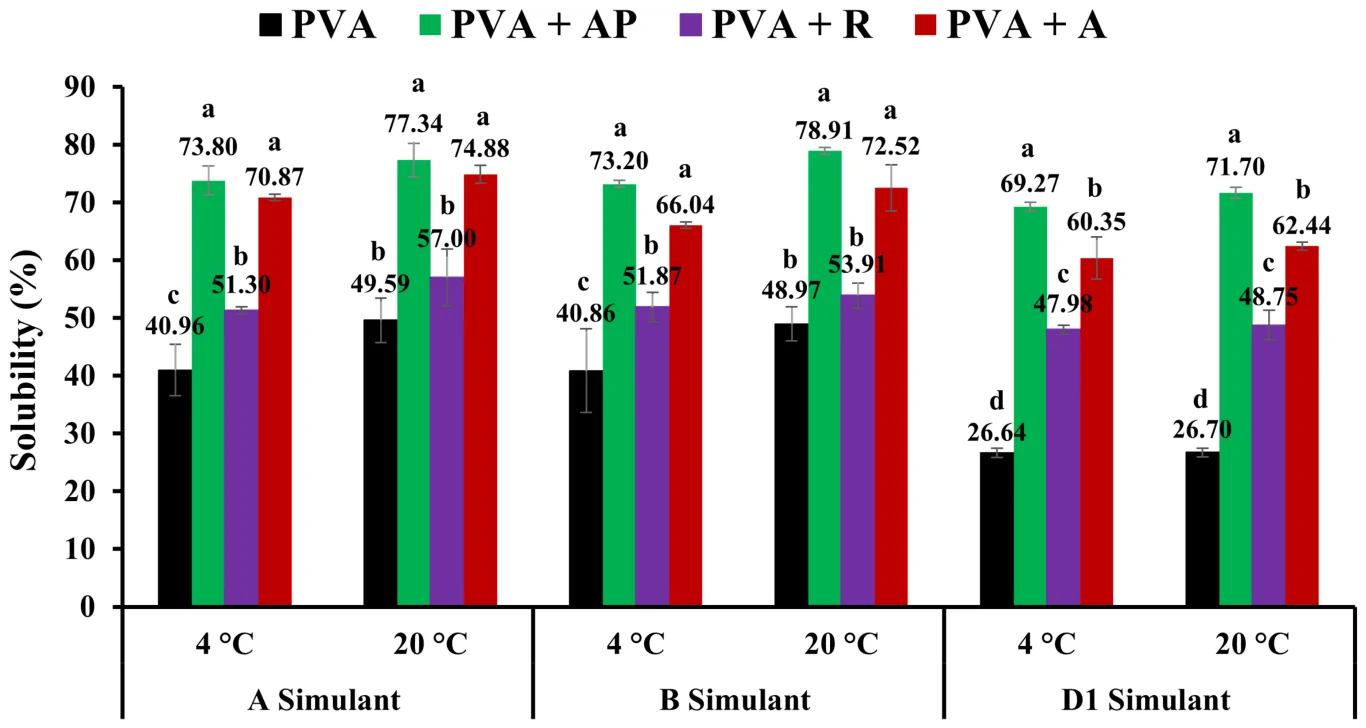
Solubility in food simulants of prepared films, where A—aronia; AP—apple peel; R—rutin. According to ANOVA One Way, using the Tukey model, when the same letter is used, there is no statistically significant difference between values. Different letters indicate that the difference between values is statistically significant at a level of more than 95%.

Solubility is an important feature of a packaging material as it can cause loss of packaging integrity as well as the migration of bioactive compounds into food [76]. The solubility values of the films obtained can be observed in Figure 12.

All three modified films showed higher solubility in the food simulants tested than the reference film, with significant statistical differences between the values. The PVA + AP film exhibited the highest solubility in the tested simulants, followed by the PVA + A film, which did not show significant statistical differences in simulations A and B. This could be due to the high affinity of apple and aronia extracts with water, as well as the higher polarity of acetic acid compared to ethanol thus facilitating the dissolution of films. These results could be correlated with the large number of C-OH bonds of the PVA + A and PVA + AP films as evidenced by the C1s deconvolution in the XPS analysis. A higher solubility of PVA + A film is due to the glycation of anthocyanins present in the structure of the aronia [77]. The solubility values in simulant D1 differed statistically significantly for all films. The PVA + R film exhibited the lowest solubility in all three food simulants, with the lowest results in the D1 simulant due to insolubility in water and poor solubility in ethanol. The films were more soluble in simulants tested at 20 °C than at 4 °C. These results are consistent with WVP analysis, the PVA + R film recording the lowest values, and with XRD analysis, due to the more orderly arrangement of the network. The PVA + R film could be used as a potential packaging material for aqueous, acidic and fatty foods and the PVA + A film could represent a potential packaging for high-fat foods. Similar results were obtained by Sun et al. [77], who indicated that chitosan films with the addition of apple polyphenols showed greater solubilities in water as the percentage of polyphenols in the film matrix increased, due to hydrophilic groups of phenolic compounds that can easily bind to water molecules. Van Nguyen and Lee [29] prepared modified PVA films with cellulose nanocrystals (CNC), titanium dioxide, and apple peel extract (APE), indicating that the release of APE in a 50% ethanol solution was higher for the PVA/CNC/APE film as compared to the PVA/CNC/TiO2/APE film. Also, Narasagoudr et al. [33] showed that adding an adequate amount of rutin to the PVA and chitosan film reduced the water solubility of the composite film due to the restriction of polymer chain mobility by hydrogen bonds, leaving few hydroxyl groups available to bind the water molecules.

### 3.11. Thermo-Welding Feasibility

All the films tested were thermo-welded. Similar results were obtained by Arora et al. [78], which indicated the high heat seal ability of the polyvinyl alcohol film reinforced with pea hull.

### 3.12. Changes of Tensile Strength 

**Figure 13:**
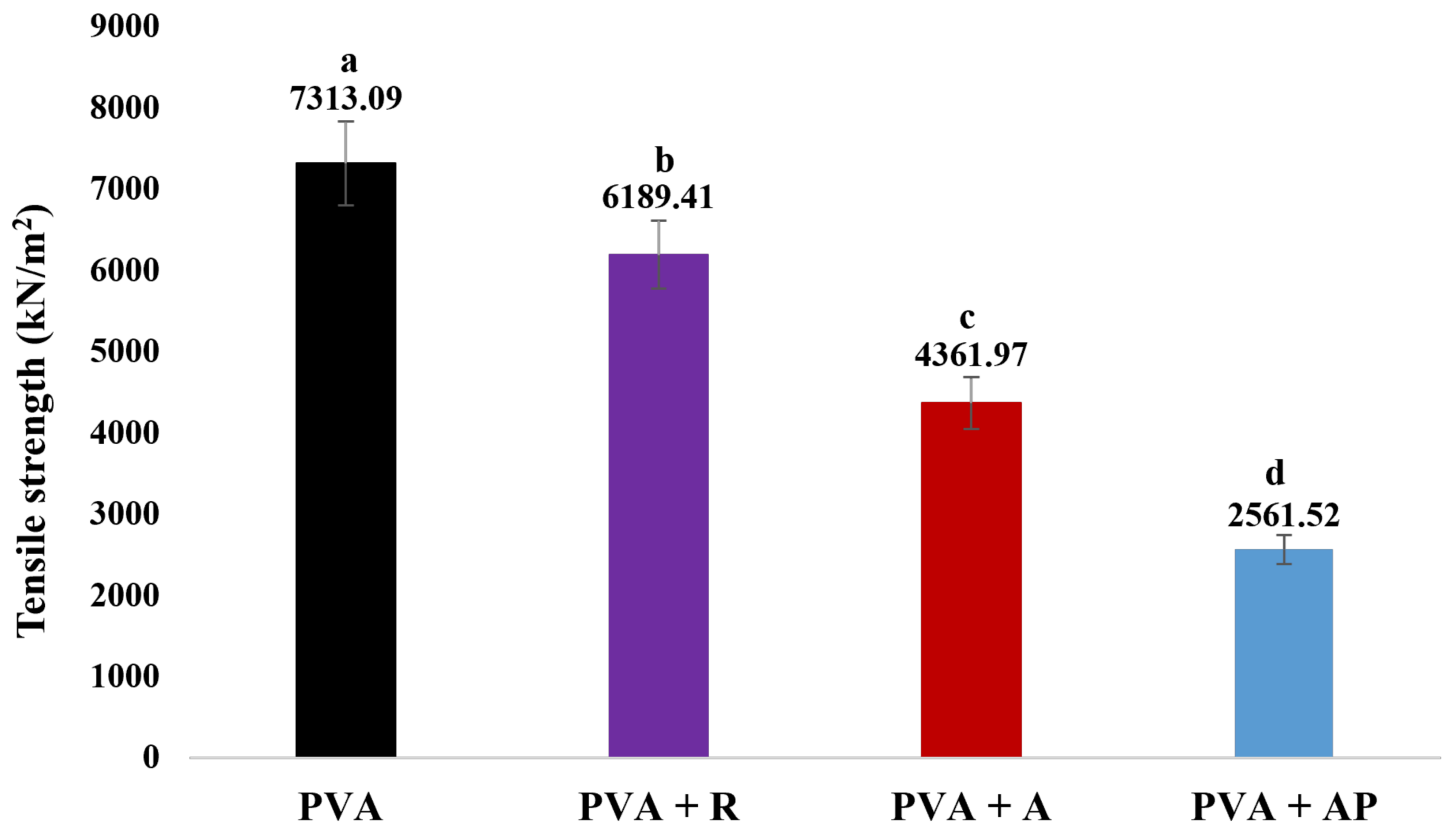
Tensile strength of prepared films, where R—rutin; A—aronia; AP—apple peel. According to ANOVA One Way, using the Tukey model, different letters indicate that the difference between values is statistically significant at a level of more than 95%.

Tensile strength is an important feature of packaging because it can prevent the spoilage of packaged products and ensure protection against external factors. The tensile strength values recorded by the prepared films can be seen in Figure 13.

Adding polyphenolic compounds in the PVA matrix led to lowering the tensile strength of the films obtained, all of which recorded values lower than the reference film, with statistically significant differences between them and the PVA film. The best values for the reference film were recorded by the film PVA + R with 6189.41 kN/m^2^, followed by PVA + A with 4361.97 kN/m^2^ and PVA + AP with 2561.52 kN/m^2^. This phenomenon could be the result of the intercalation of phenolic compounds in the PVA matrix, for which the data is supported by the results of the structural analyses, which led to a weakening in mechanical strength.

### 3.13. Variation of Antioxidant Activity 

**Figure 14:**
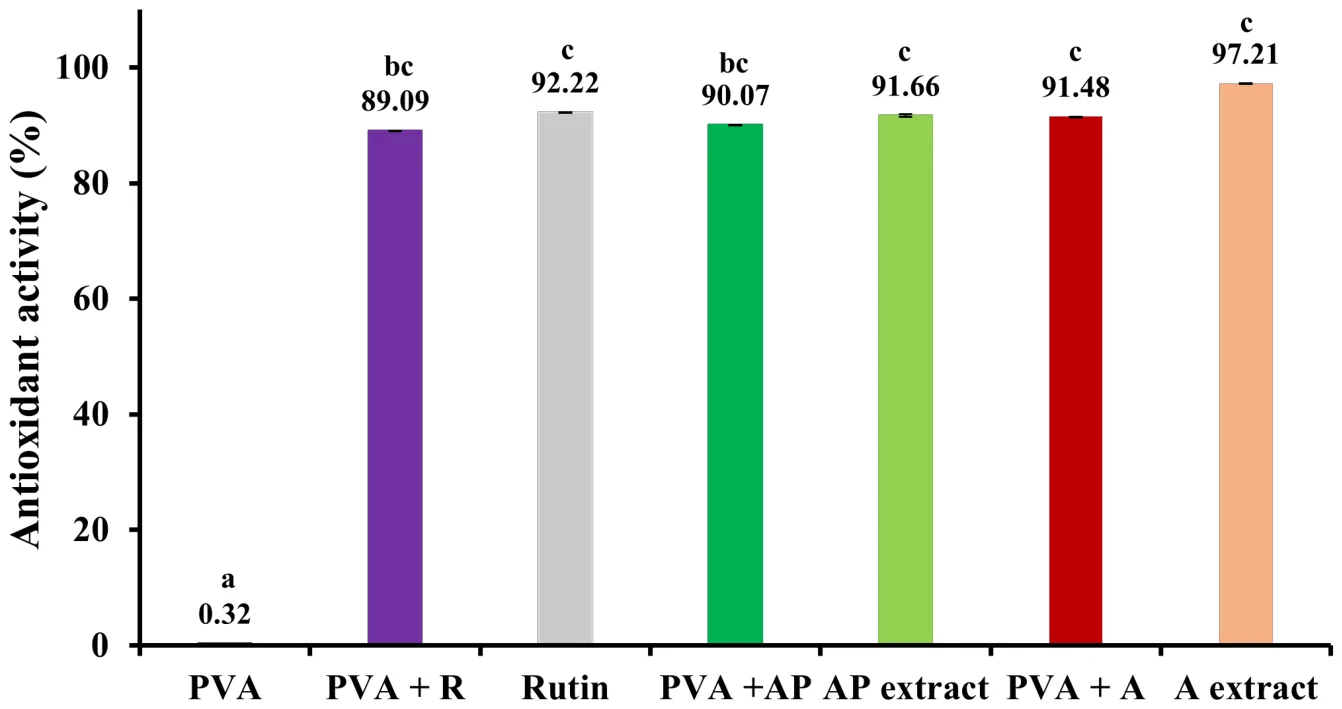
Antioxidant activity of extracts and films obtained, where A—aronia; AP– apple peel; R—rutin. According to ANOVA One Way, using the Tukey model, when the same letter is used, there is no statistically significant difference between values. Different letters indicate that the difference between values is statistically significant at a level of more than 95%. When both letters are present, it suggests that there are no statistically significant differences between values.

The antioxidant activity of packaging films is an important feature for packaging material because it can prevent or delay the oxidation of fats, which would lead to damage to food and loss of its nutritional quality, with the effect of forming potentially harmful compounds, discoloration of products and the formation of unpleasant odors [24]. The ability to capture DPPH radicals is an important method to assess the antioxidant properties of the film obtained. The antioxidant activity of the extracts and films obtained can be seen in Figure 14.

The PVA film featured very low DPPH radical scavenging activity, namely 0.32%. All extracts introduced in the reference film indicated strong antioxidant activity, the best results being obtained by the aronia extract with a free radical scavenging activity of 97.21%, followed by rutin with 92.22% and apple peel extract with 91.66%. There are no statistically significant differences between these values. All the films modified with phenolic compounds significantly improved the antioxidant activity of PVA film by about 90%; the best results are achieved by the PVA + A film with a free radical scavenging activity of 91.48%. This would be due to the strong antiradical activity of the phenolic components present in the film, attributed to the highly reactive hydroxyl groups. The activity of capturing DPPH radicals is achieved either by electron donation, by donating the electron reducing the radical, or by hydrogen donation, thus inhibiting the free radical [79,80]. Antioxidants responsible for the antiradical activity of rutin, which plays an important role in capturing free radicals, are dysaccharide rutinosis and quercetin flavonol [32]. The high antiradical activity of aronia would likely be due to phenolic acids that facilitate access to the radical site due to their small size causing the rapid and easy capture of free radicals DPPH [25]. The antiradical activity of the apple peel is mainly due to the presence of procyanidins, quercetins, chlorogenic acids, and catechins, which release the hydrogen ion bound to the aromatic ring structures, which combine with the free radicals DPPH, forming stable intermolecular bonds [2]. These findings align with the FTIR, SEM, and XPS analyses, indicating a high density of intermolecular and interfacial bonding within the modified film matrices. These data are consistent with those present in the literature, namely Roy and Rhim [15], which indicated that the addition of cinnamon essential oil and the rutin to the films based on chitosan and gelatin significantly improved their antioxidant activity. Also, Oun et al. [13] prepared PVA and chitosan-based films with the addition of aronia, cellulose nanocrystals and grapefruit seed extract, and reported that the procyanidins in the composition of aronia would be responsible for the high antioxidant activity that the modified aronia films exhibited. Ferreira et al. [26] studied the effect of different storage conditions on the phenolic composition and antioxidant activity of two varieties of apples, namely Red Delicious and Golden Delicious, and indicated that procyanidin B2, anthocyanins, (-)-epicatechin, and total phenols positively contributed to the antioxidant activity of apple peel in both varieties.

### 3.14. Variation of Antimicrobial Activity 

**Figure 15:**
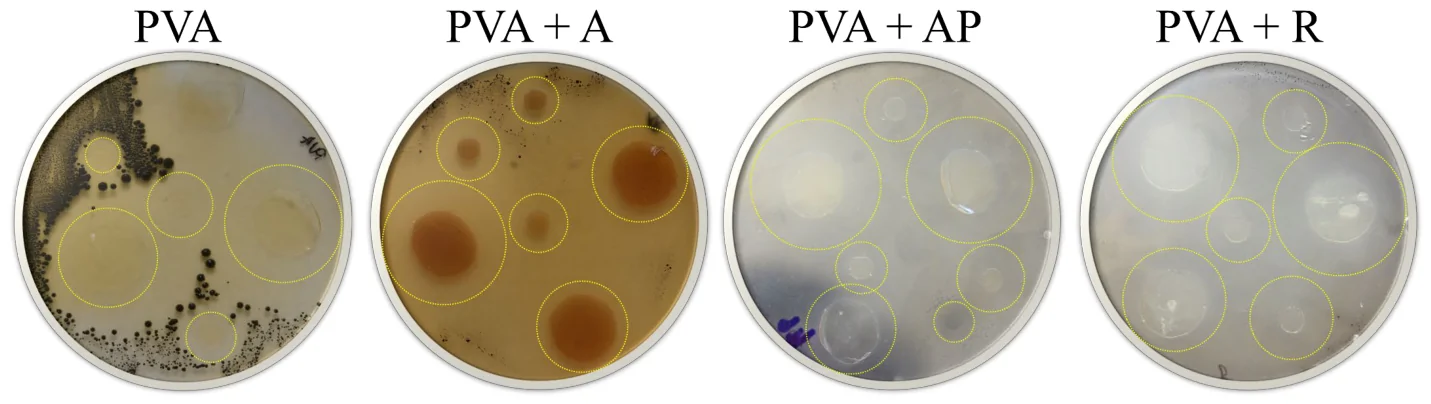
Macroscopic images of the microbiological samples of the film obtained inoculated with S. aureus, after 48 h of incubation, at 37 °C, where A—aronia; AP—apple peel; R—rutin.

The antimicrobial properties of films prepared against *S. aureus*, a food-borne pathogen that could cause various infections to humans, were assessed by measuring inhibition areas. The results obtained can be seen in Figure 15.

The PVA film demonstrated an antimicrobial effect against *S. aureus*, evidenced by the formation of an inhibition zone ranging between 3 mm for small circles and 10 mm for large circles, probably due to the presence of CA in the film’s matrix, which creates an acidic environment, lowering the local pH, inhibiting the growth of bacteria. In the PVA + AP and PVA + R films, the fewest colonies of *S. aureus* developed; these were isolated at the edge of the Petri dish, being round, convex and gray to black [81]. These films thus prevented the growth of bacteria in the areas between the circles, showing a clean surface. The PVA + AP and PVA + R films showed a 10 mm inhibition area around small circles and 20 mm around large circles. The mechanism of action would consist of altering cytoplasmic membranes and the cell wall, thus facilitating access to different layers, causing their structure to be destroyed [82]. Similar results were obtained by Horvacki et al. [83] and Van Nguyen and Lee [29], who indicated that the antimicrobial activity of apple peel extract may be due to the action of flavonoids on certain proteins located on the surface of the cytoplasmic membrane, namely the components of peptidoglycan, leading to cell death. Furthermore, PVA films incorporating rutin have been reported to exhibit significantly enhanced antimicrobial activity compared to reference films, as evidenced by larger inhibition zones against *S. aureus*. This effect is generally attributed to the sustained availability of rutin within the polymer matrix, which promotes prolonged interaction with bacterial cells and leads to membrane perturbation and impairment of essential cellular functions [33,84]. The PVA + A film showed higher antibacterial activity than the reference film, with inhibition areas around the circles ranging between 5 and 7 mm for small circles and 17 and 20 mm for large circles, probably due to the presence of anthocyanins and phenolic acids in the composition of aronia, which causes destabilization of the cytoplasmic membrane and has an inhibitory effect on the secretion of extracellular enzymes. These results are supported by Long et al. [23] and Koosha and Hamedi [68], which indicated that the mechanism of action of polyphenols added in the modified films would be due to the permeation of cell membrane, thus preventing the release of the substrate necessary for bacterial growth and extracellular enzyme secretion.

All the films obtained showed an inhibitory effect against the Gram-positive bacterium *S. aureus*, although the extent and clarity of the inhibition zones varied among the formulations, with values between 0.2 and 0.7 mm for small circles and 7 and 10 mm for large circles. The incorporation of polyphenols into the PVA matrix was associated with an increased inhibitory effect compared with the PVA-based formulation. However, based on the inhibition zone assay alone, the contribution of the individual components and any possible synergistic interactions cannot be conclusively established. The observed antimicrobial effect may be related to interactions between the phenolic compounds and bacterial cell membranes, potentially affecting membrane integrity and permeability.

### 3.15. Mechanism of Release of Polyphenols from the Films Obtained

**Figure 16:**
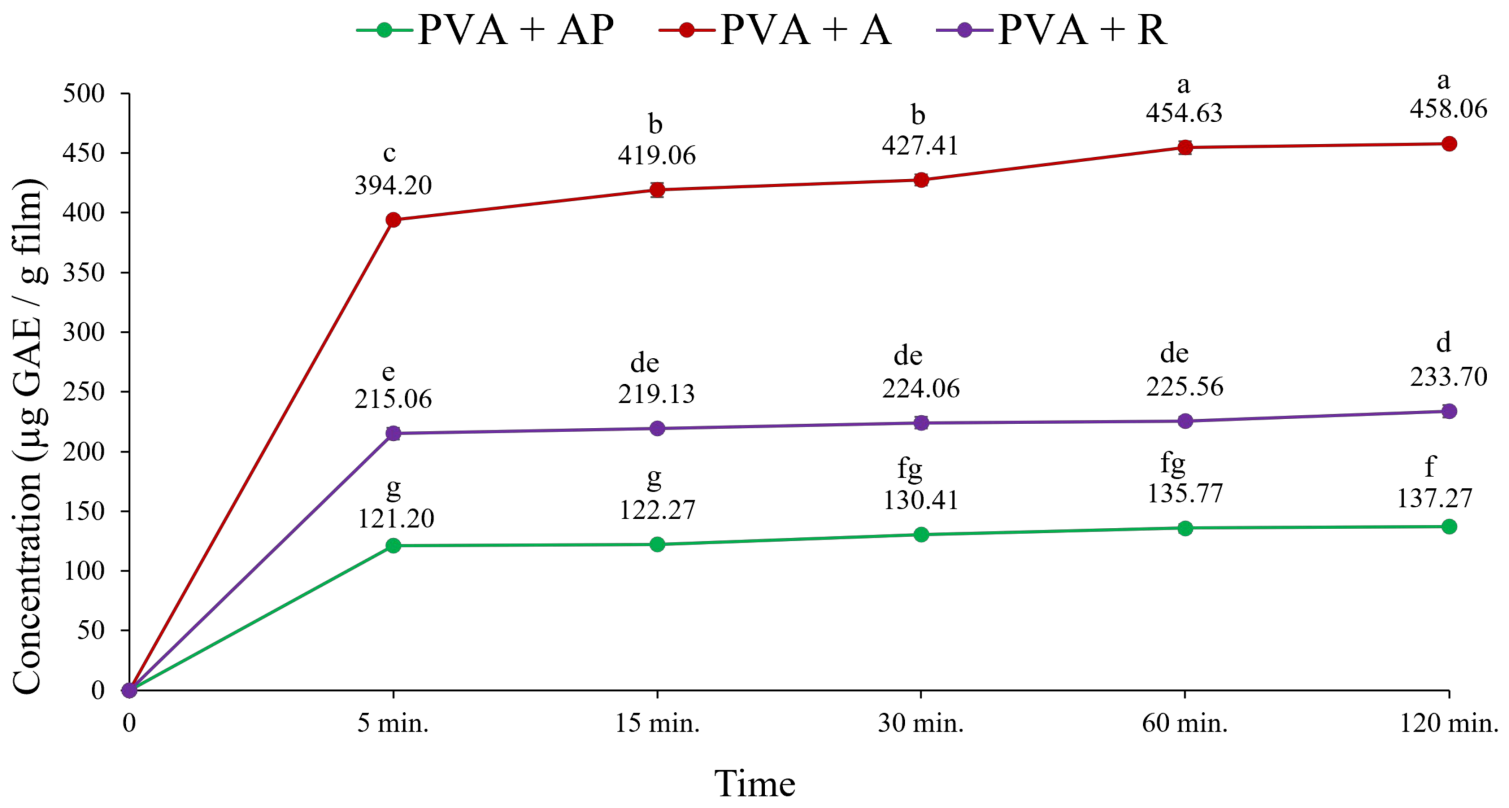
The values of polyphenols released from the films obtained, where A—aronia; AP—apple peel; R—rutin. According to ANOVA One Way, using the Tukey model, when the same letter is used, there is no statistically significant difference between values. Different letters indicate that the difference between values is statistically significant at a level of more than 95%.

The total polyphenol content (TPC) of the extracts was 1.85 mg GAE/g film for AP, 5.51 mg GAE/g film for A and 1.28 mg GAE/g film for R. The release of polyphenols from films increases over time, but in the first 5 min the amount of polyphenols released was more than 85% for all three films, as shown in Figure 16 and Table 3. The PVA + A film released the largest amount of polyphenols, with values ranging from 394.20 to 458.06 µg GAE/g film across five measurements. The content of polyphenols released by the PVA + R film ranged from 215.06 to 233.7 µg GAE/g film, while the amount released by the PVA + AP film ranged from 121.2 to 137.27 µg GAE/g film. The data obtained are consistent with the HPLC analysis, which indicates that aronia extract contained a greater quantity of polyphenols than apple peel extract. Comparable behavior was reported by Zhang et al. [85], who investigated the release of liposoluble tea polyphenols from functional edible films composed of hydroxypropyl methylcellulose, sodium citrate, and liposoluble tea polyphenol/water Pickering emulsions stabilized with starch nanoparticles. Their study showed a rapid initial release phase followed by a slower, sustained release over time.

The phenolic release profiles of PVA-based films highlight a kinetics characterized by a rapid release effect in the first 5 min followed by a slow plateau phase up to 120 min.

(i) Initial rapid release phase (0–5 min): In the first 5 min there is a release of more than 86% of the total quantity of polyphenols (86.1% for PVA + A, 88.3% for PVA + AP and a maximum of 92% for PVA + R). This phenomenon is attributed to the almost instant desorption of phenolic compounds located on the surface of films or in their superficial layers.
(ii) Whole-range modelling (0–120 min): The kinetic model of Order I describes the experimental data excellently (R^2^ ≥ 0.990 for all formulations). The high speed constants (k_1_ = 0.449–0.611 min^−1^) mathematically reflect the high speed of the initial mass transfer generated by the steep concentration gradient. The calculated theoretical maximum capabilities (C_max calc._) are fully consistent with the experimentally measured maximum values. The kinetic model of Order I can be seen in Table 4.

(iii) The slow-phase diffusion mechanism (5–120 min): Since the initial release exceeds the 60% threshold required by the classic Korsmeyer–Peppas model, the secondary plateau phase was assessed by the Higuchi model. The good linear dependence in relation to square root of √t (R^2^ = 0.849–0.964) demonstrates that the residual migration of bioactive compounds captive to the depth of the PVA polymer network is given exclusively by a Fickian diffusion mechanism without matrix decay. Low diffusion rates Higuchi (*k_H_* ≈ 2.04 − 2.05 μg ⋅ g^−1^ ⋅ min^−0.5^ pentru PVA + AP și PVA + R) attests to the ability of films to effectively retain and stabilize the internal fraction of antioxidants. The data of Higuchi model can be seen in Table 5.

### 3.16. Applicability of Films as Packaging Materials for Butter Storage

#### 3.16.1. Variation of Gravimetric Dynamics of the Butter-Package System During Long-Term Storage

**Figure 17:**
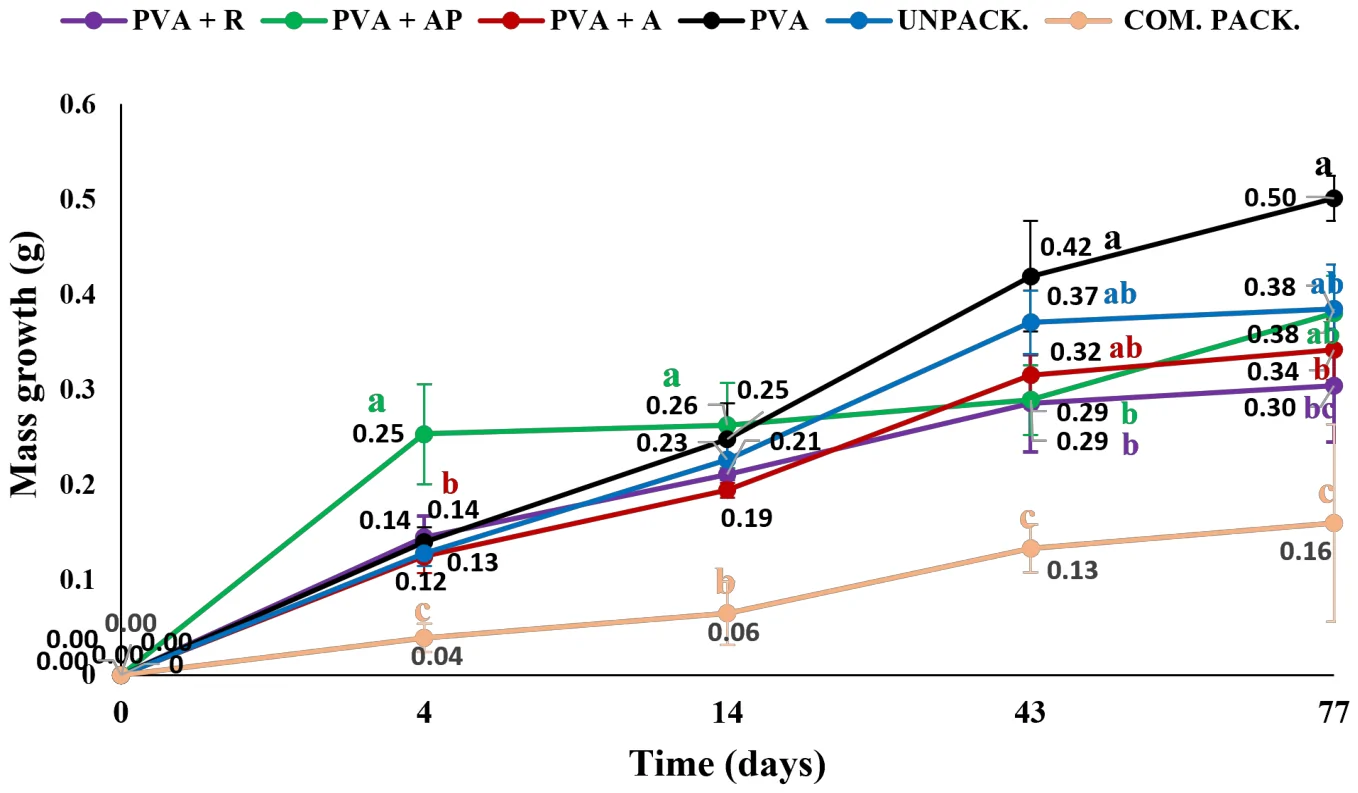
Mass growth of butter packed in films produced, in commercial packaging (COM. PACK) and unpacked (UNPACK.) for 77 days in refrigeration conditions. Where A—aronia; AP—apple peel; R—rutin. According to ANOVA One Way, using the Tukey model, when the same letter is used, there is no statistically significant difference between values. Different letters indicate that the difference between values is statistically significant at a level of more than 95%. When both letters are present, it suggests that there are no statistically significant differences between values.

Analysis of the mass variation of the complete system (butter + package) revealed the statistically significant differences (*p* < 0.05) between the samples tested, directly reflecting the permeability and hydrophilic character of the materials used, as can be seen in Figure 17.

The system protected with PVA films recorded the highest mass growth (0.50 g, statistical group a). This behavior would be due to the high density of hydroxyl groups present in the PVA network, which massively absorbed moisture and water vapor from the atmosphere of the refrigerator, functioning as a hygroscopic absorber. The incorporation of natural polyphenols has reduced this trend of water accumulation. The PVA + R system increased by 0.30 g (bc statistical group) and PVA + A system by 0.34 g (statistical group b), both of which were significantly more stable than PVA film. This behavior demonstrates that the presence of flavonoids and anthocyanins, through potential intermolecular hydrogen bonds formed with functional groups of the PVA films (observed in FTIR analysis), partially blocks the hydrophilic sites of the polymer, reducing its ability to absorb water from the outside. The samples packaged in the PVA + AP films and unpacked samples (UNPACK.) showed a variation of 0.38 g (statistical group ab). These results are consistent with the values of water absorption, solubility and WVP, where the PVA + AP film obtained the highest values. In the case of unpacked sample, the change is an imbalance caused by dehydration and the formation of an oxidized crust on the surface, which is associated with the intense yellowing observed visually on day 77. Commercial packaging ensured the highest gravimetric stability of butter, registering a minimum variation of 0.16 g (statistical group c), statistically comparable to PVA + R and PVA + A films on day 77. This result confirms the ideal hydrophobic behavior of the industrial material, which blocks the transfer of water vapors from the atmosphere of the refrigerator to the food and vice versa.

All the butter samples showed a uniform yellowish white color in the whole mass, with characteristic gloss on the surface, unctuous, compact, homogeneous and unbreakable consistency. Differences arose on day 77 in terms of color and gloss, the butter having a more yellowish color and losing a bit of its characteristic gloss on the surface but keeping these characteristics in cross-section. In the cross-section, the butter presented a continuous surface, without air gaps or visible water droplets. The smell was pleasant, characteristic of fresh butter, without foreign shades of flavor. Visual examination after 77 days of storage revealed a clean and homogeneous appearance, with no visible microbial growth or other foreign substances on the surface or in the section.

#### 3.16.2. Kinetic Profile of Oxidative Degradation and Structural Stability of Biofilms over 77 Days

**Figure 18:**
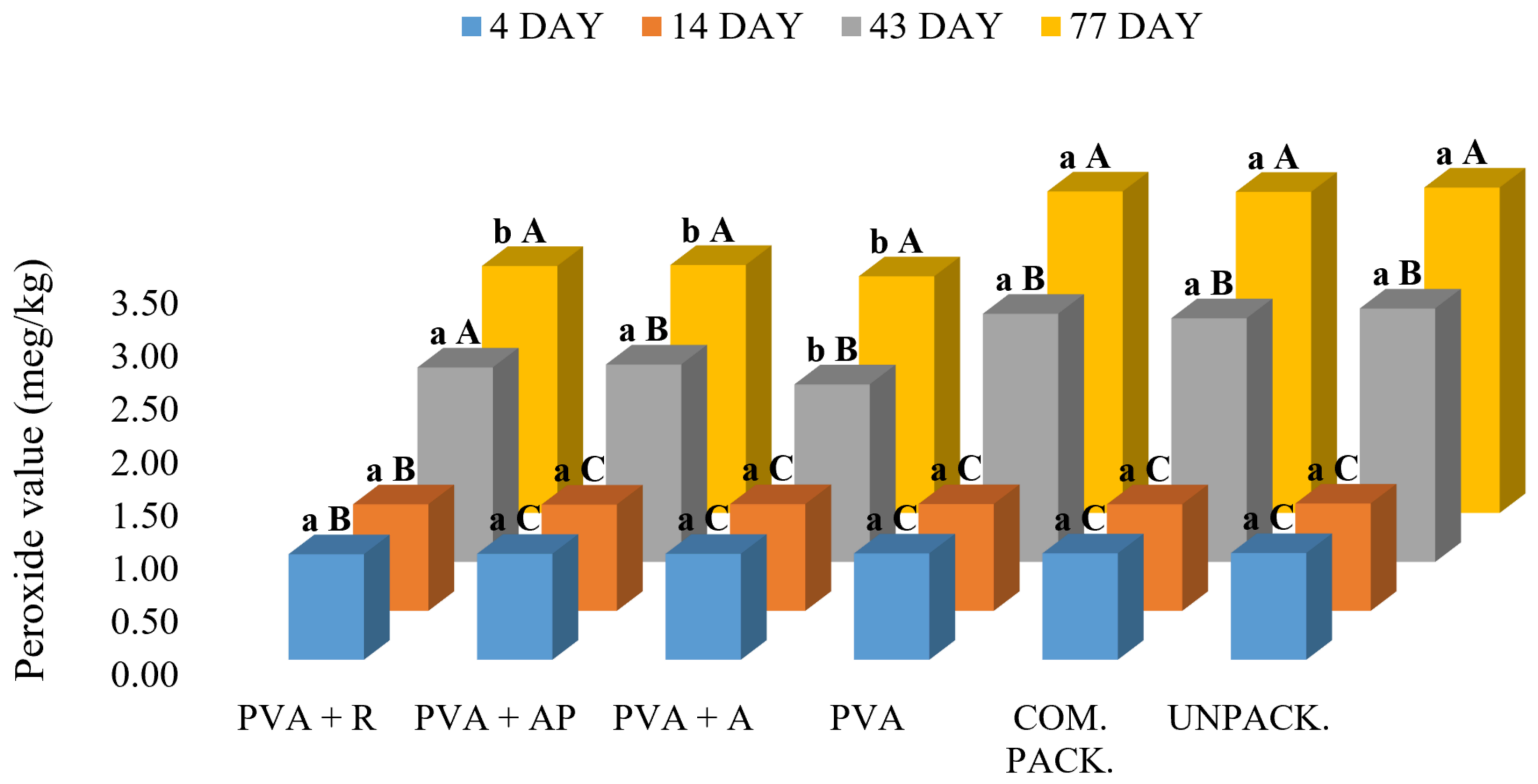
PV (meq/kg) recorded by butter packaged or unpackaged. Where A—aronia; AP—apple peel; R—rutin; C. PACK.—commercial packaging; UNPACK.—without packaging. According to ANOVA Two Way, using the Tukey model, different letters indicate that the difference between values is statistically significant at a level of more than 95%. Small letters—differences between films at the same time interval; caps letters—differences between time intervals for the same film.

The monitoring of butter at the established intervals of 4, 14, 43 and 77 days allowed for the mapping of the oxidative degradation mechanism and the behavior of the polymer barrier. The PV registered of the tested butter can be seen on Figure 18.

**Figure 19:**
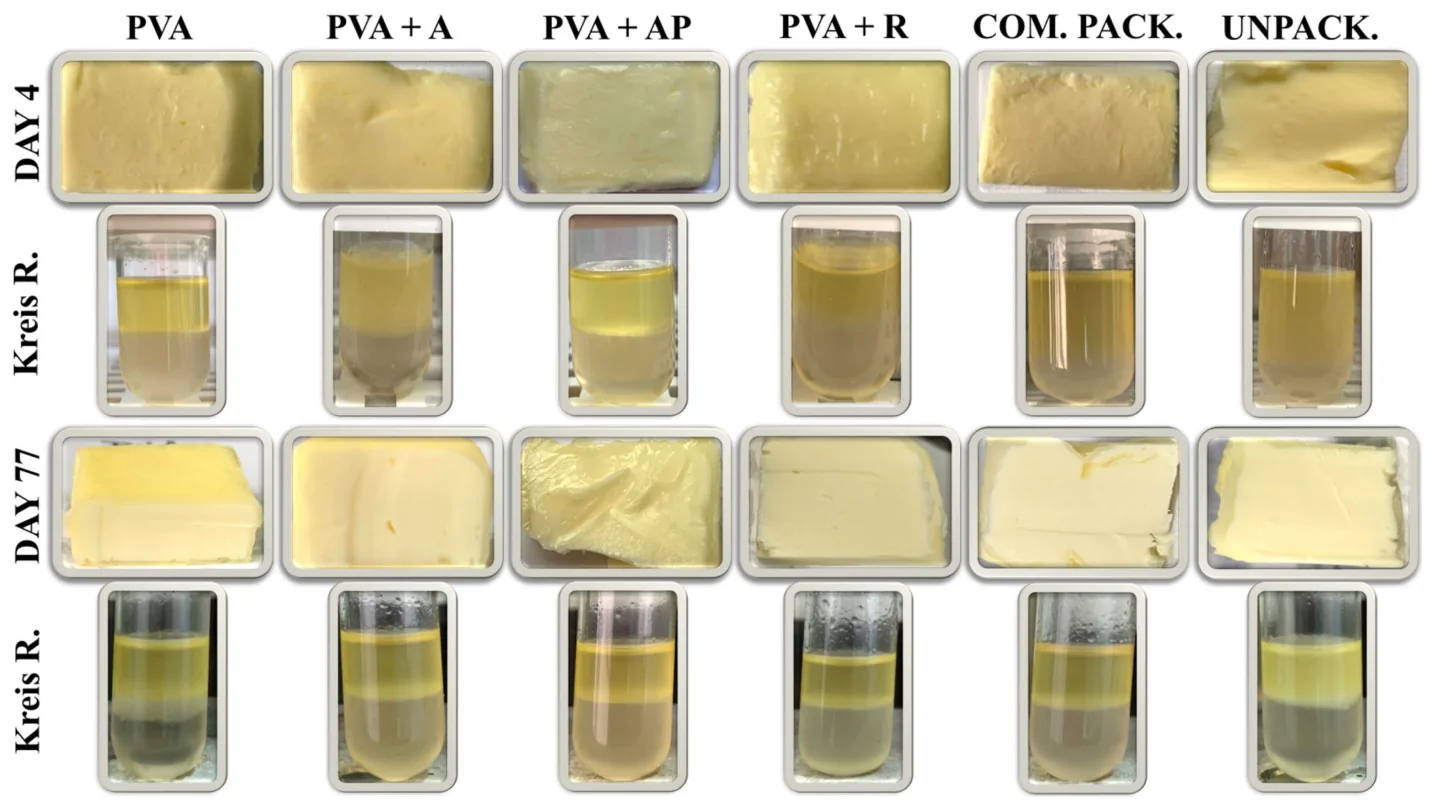
Macroscopic images of the monitored butter on day 4 and day 77, and the results of the Kreis R. Where A—aronia; AP—apple peel; R—rutin; COM. PACK.—commercial packaging; UNPACK.—without packaging.

During the first days of storage (days 0 and 14), the PV recorded minimal basal values. In this period, the biofilms are balanced hydrophilically with the surface of the butter, reaching a controlled degree of swelling (registered as stable mass growth). The critical inflection point arose on day 43, at which time the reference film and UNPACK samples began to accumulate primary oxidation products at an accelerated rate, indicating leaving the induction phase and entering the propagation phase. During this time, modified polyphenol biofilms maintained extremely low PV levels, except for PVA + AP, in line with TPC initial and HPLC analysis, indicating lower polyphenol content. A physical network controlled exclusively by hydrogen bonds (identified by FTIR) acted as an intelligent release system: the moisture taken from the butter and the storage medium generated a relaxation of the PVA chains, facilitating the continuous migration of the antioxidant molecules (anthocyanins from aronia, quercetin/rutin from the apple peel and pure rutin) directly from the butter surface. The maximum antioxidant efficiency was quantified on day 43, where PVA + A demonstrated the most robust protection (PV = 2.03 meq/kg), followed by PVA + R (PV = 2.23 meq/kg) and by C. PACK (PV = 2.46 meq/kg). The PVA + AP film recorded PV values slightly higher (PV = 2.57 meq/kg). However, the extension of storage up to day 77 highlighted the physical limits of the thermal untreated network. The passage of time from day 43 to day 77 triggered an intense, macroscopically visible yellowing of the butter surface, more pronounced in the reference samples and butter unpacked and partially present in the developed packaging. This change in the color and luster characteristic of butter signals severe surface dehydration doubled by the oxidation termination phase, where hydroperoxides break down into secondary compounds (aldehyde, ketones). The results of the Kreis reaction sustain the results of the PV indicating a slight coloring to pink. Macroscopic images of the tested butter and the Kreis reaction results can be seen in Figure 19.

From a kinetic point of view, the developed biofilms provide an excellent active protection period of up to 43 days, after which the absence of a rigid covalent barrier allows for mass transfer of water vapors and visual degradation of the food.

The presence of polyphenols in the structure of the PVA matrix prevented oxidative processes during the storage of butter in refrigeration conditions for 43 days and it maintained the quality of butter, probably due to their free radical scavenging ability, which could cause oxidation and rancidity phenomena. These results are in line with the indicated antioxidant and antimicrobial activity of the modified films. The PVA + R and PVA + A films could be reliable candidates for fatty food packaging, which are prone to oxidation and rancidity. Similar results were obtained by Wang et al. [85], who prepared films with gelatin and anthocyanin nanocomplexes and tested their effectiveness in preserving olive oil for 56 days. The authors of the study recommend functional films obtained as a potential packaging material capable of delaying oxidation processes and prolonging the shelf life of fatty foods. Also, Alshehri et al. [14] prepared PVA and carboxymethyl cellulose composite films with the addition of broccoli sprout seed extract in various concentrations and tested the butter packing films for 50 days. Analyses showed that the acidity of the butter packaged in these films was not substantially altered during storage, with the authors attributing this to reducing the moisture content of films, thus also reducing the hydrolysis of triglycerides. The authors also assessed the film’s antioxidant activity through TBARS analysis to study how it affects the secondary oxidation of butter and concluded that the modified films extended the shelf life of butter, due to the presence of hydroxyl and carboxyl groups in the matrix structure, by capturing free radicals and thereby reducing the amount of primary oxidation compounds, such as peroxides, which could be formed, thus preventing the occurrence of undesirable phenomena of secondary oxidation. Following the analysis of the peroxide index, the modified films demonstrated the oxidative stability effects on butter during storage, the authors attributing this to the presence of antioxidant compounds inside the matrix and the opacity and thickness of the films, thus delaying oxidation and prolonging the butter retention period. The authors of this study recommend modified films as potential packaging materials for a variety of food applications.

## 4. Conclusions

The purpose of this research was the modification of the PVA film with aronia extracts, with apple peel extracts, and rutin to obtain a potential packaging material with functional properties, which could be used to preserve the quality and safety of foods with high oxidation potential.

Incorporating polyphenols into the PVA matrix promotes intermolecular and interfacial interactions through hydrogen bonds supported by EDXS, FTIR and XPS analysis, as well as the increased structural order indicated by XRD analysis. SEM and AFM analyses indicated the successful integration of polyphenols in the PVA matrix by the presence of homogeneous surfaces, no interfacial phase separations, and small morphological differences between the films. All the films obtained exhibited very low-fat permeability. The water barrier properties of the PVA film were not influenced by the polarity of the phenolic compounds and results comparable to it were obtained, except PVA + AP film. The PVA + A film demonstrated a UV-Vis light shielding capacity of 47%. All modified films had higher solubility in the simulants tested than the PVA film, due to poor water resistance of phenolic components. Modified films with polyphenols demonstrated double bio-functionality, improved the antioxidant properties of the reference film by about 90%, demonstrating their strong ability to alleviate oxidative stress, and exhibited enhanced antimicrobial activity compared to the reference film against the Gram-positive bacterium *S. aureus.* These results suggest that the incorporation of polyphenols into the PVA matrix may contribute to preserving butter quality during storage. The developed active films demonstrated promising applicability in butter packaging tests, maintaining the quality of butter for up to 43 days. The packed butter showed no visible signs of oxidation or rancidity. Based on visual examination, the surface remained clean, with no visually detectable signs indicative of microbial development.

The findings of this study demonstrate that the developed active films successfully fulfilled their intended objectives. The authors suggested that PVA + A and PVA + R films could have considerable potential as packaging materials for fatty foods that are highly susceptible to oxidative degradation and that PVA + AP films could represent promising candidates for packaging applications involving high-moisture foods.

Future research could be aimed at exposing biofilms to gentle heat treatment (for example, 80 °C for 2–3 h), without degrading the thermosensitive polyphenols. This could trigger a partial esterification reaction between citric acid, polyphenols and PVA, creating a much tighter polymer network that allows for a slower release of polyphenols, providing prolonged protection. Viewed as a whole, this research offers a reliable strategy for obtaining active PVA-based packaging materials for food packaging, highlighting the potential of agricultural by-products in the context of circular bioeconomy.

## Figures and Tables

**Table 1:** AFM data of the films obtained, where A—aronia; AP—apple peel; R—rutin. According to ANOVA One Way, using the Tukey model, when the same letter is used, there is no statistically significant difference between values.

Film	Average Roughness S_a_ (nm)	Root Mean Square S_q_ (nm)	Average Particle Size/Diameter (nm)
PVA	0.63 ± 0.02 ^a^	0.82 ± 0.02 ^a^	15.7 ± 0.06 ^a^
PVA + R	0.68 ± 0.02 ^a^	0.85 ± 0.01 ^a^	17.85 ± 0.01 ^a^
PVA + A	1.01 ± 0.01 ^a^	1.26 ± 0.01 ^a^	25.5 ± 0.04 ^a^
PVA + AP	1.06 ± 0.01 ^a^	1.35 ± 0.02 ^a^	19.9 ± 0.05 ^a^

**Table 2:** The deconvolution data of XRD spectra. Different letters indicate that the difference between values is statistically significant at a level of more than 95%.

Sample/Formulation	*xc*_crystalline_ (o)	*A_c_* (Crystalline Area)	*A_a_* (Amorphous Area)	Crystallinity Index (CI, %)	R^2^	Structural State
PVA	-	0.00 ± 0.00	100.00 ± 0.00	0.00 ± 0.00 ^d^	-	Completely amorphous
PVA + AP	19.93	2071.91 ± 22.61	9969.97 ± 69.37	17.21 ± 0.39 ^c^	0.967	Semi-crystalline
PVA + R	19.92	2282.27 ± 19.31	9834.99 ± 65.26	18.83 ± 0.24 ^b^	0.960	Semi-crystalline
PVA + A	20.07	2904.09 ± 26.29	11,677.10 ± 81.65	19.92 ± 0.48 ^a^	0.969	Semi-crystalline
R—powder	19.88	2187.64 ± 21.01	8928.24 ± 96.54	19.68 ± 0.41	0.961	Semi-crystalline
A—powder	-	0.00 ± 0.00	100.00 ± 0.00	0.00 ± 0.00	-	Completely amorphous
AP—powder	-	0.00 ± 0.00	100.00 ± 0.00	0.00 ± 0.00	-	Completely amorphous

**Table 3:** The release of polyphenols from the films.

Sample	TPC Initially (mg/g Film)	TPC Released (mg/g Film)	Release (%)	Retained (%)
PVA + AP	1.85	0.137	7.41	92.59
PVA + A	5.51	0.458	8.31	91.69
PVA + R	2.28	0.233	10.21	89.79

**Table 4:** Data by Order I kinetic model (0–120 min).

Film	C_max exp._	C_max calc._	Model Order I: *k*_1_ (min^−1^)	Model Order I: R^2^ (COD)	% Release at 5 min (Burst)
PVA + AP	137.27 ± 2.12	131.49 ± 2.94	0.506 ± 0.124	0.990	88.30 ± 2.13
PVA + R	233.70 ± 5.15	225.63 ± 2.62	0.611 ± 0.109	0.997	92.00 ± 2.75
PVA + A	458.06 ± 2.42	440.08 ± 8.42	0.449 ± 0.079	0.993	86.00 ± 2.49

*C*_max exp._: Maximum actual quantity extracted from the film at the end of the test (at 120 min). *C*_max calc._: The theoretical maximum capacity at the plateau predicted by the mathematical equation. *k*_1_: The speed constant of the release process—the higher it is, the more sudden the initial discharge. *R*^2^: How faithfully the theoretical curve follows the experimental points. % Burst: Percentage released almost instantly at 5 min by surface desorption.

**Table 5:** Data by Higuchi model (5–120 min).

Film	*C*_0_ (μg/g)	*k_H_* (mg ⋅ g^−1^ ⋅ min^−0.5^)	Pearson’s *r*	*Adj*. *R*^2^	Predominant Transport Mechanism
PVA + AP	116.94 ± 2.88	2.055 ± 0.425	0.941	0.955	Fickian diffusion limited by matrix
PVA + R	211.15 ± 1.34	2.039 ± 0.197	0.986	0.960	Fickian diffusion limited by matrix
PVA + A	386.27 ± 9.91	7.331 ± 1.462	0.945	0.964	Fickian diffusion limited by matrix

*C*_0_ (Intercept): Start concentration of the slow phase (corresponds to the amount already accumulated at minute 5). *k_H_* (Slope/slope): The Higuchi constant; it represents the real speed at which it diffuses the molecules locked deep into the polymer network of PVA to the solution. Pearson’s r/Adj. R^2^: Linearity indicators; confirms dependence on square root of time (√t). Deduced mechanism: Demonstrates that the slow passage of polyphenols on the plateau is exclusively guided by Fickian diffusion through an insoluble/reticulated matrix.

## Data Availability

Data will be made available on request.

## Supplementary Materials

The following supporting information can be downloaded at: https://www.mdpi.com/article/10.3390/polym18202519/s1, Figure S1. FTIR spectra of films produced; Figure S2. Deconvolution model XRD; Figure S3. The graphics are set according to the kinetic model of the first order, PVA + AP; Figure S4. The graphics are set according to the kinetic model of the first order, PVA + A; Figure S5. The graphics are set according to the kinetic model of the first order, PVA + R; Table S1: The O/C ratio from the SEM analysis; Table S2. Data extracted from the deconvolution of XRD spectra; Table S3. Deconvolution C1s; Table S4. PV (meq/kg) recorded by butter packaged or unpackaged. Where A—aronia; AP—apple peel; R—rutin; C. PACK.—commercial packaging; UNPACK.—without packaging. Section S1. Quantitative Determination of the Crystallinity Index (CI, %) by XRD Deconvolution. Section S2. Kinetics of polyphenol release.

## References

[1] Liu J., Cheng Y., Fang C., Zhang X., Li M., Xiang K. (2024). Novel design of highly biocompatible and dual-network hydrogel films containing apple polyphenol and its potential application in functional package. Ind. Crops Prod..

[2] Tang P., Li X., Li H., Li J., Tang B., Zheng T. (2024). Development of active film based on collagen and hydroxypropyl methylcellulose incorporating apple polyphenol for food packaging. Int. J. Biol. Macromol..

[3] Yuan X., Wei F., Wang Z., Li X., Deng Y., Shi X., Lin B. (2025). Transparent, mechanically robust and antibacterial food packaging film based on starch/PVA/citric acid/carboxycellulose nanofibers by one-pot green process. Int. J. Biol. Macromol..

[4] Mao L., Wang C., Dong Z., Yao J., Dong F., Dai X. (2025). Fabrication of polylactic acid bilayer composite films using polyvinyl alcohol based coatings containing functionalized carbon dots and layered clay for active food packaging. Ind. Crops Prod..

[5] Kusuma H.S., Sabita A., Putri N.A., Azliza N., Illiyanasafa N., Darmokoesoemo H., Amenaghawon A.N., Kurniawan T.A. (2024). Waste to wealth: Polyhydroxyalkanoates (PHA) production from food waste for a sustainable packaging paradigm. Food Chem.-Mol. Sci..

[6] Sharma S., Jaiswal A.K., Duffy B., Jaiswal S. (2020). Ferulic acid incorporated active films based on poly(lactide)/poly(butylene adipate-co-terephthalate) blend for food packaging. Food Packag. Shelf Life.

[7] Ragab H., Diab N., Obeidat S.T., Alghamdi A.M., Khaled A.M., Farea M., Morsi M. (2024). Improving the optical, thermal, mechanical, electrical properties and antibacterial activity of PVA-chitosan by biosynthesized Ag nanoparticles: Eco-friendly nanocomposites for food packaging applications. Int. J. Biol. Macromol..

[8] Deng H., Su J., Zhang W., Khan A., Mahmood M.A., Goksen G., Kashyap P., Ezati P., Rhim J.W. (2024). A review of starch/polyvinyl alcohol (PVA) blend film: A potential replacement for traditional plastic-based food packaging film. Int. J. Biol. Macromol..

[9] Thomas J., Thomas P.S., Stephen R. (2024). Improved mechanical, thermal, and barrier properties of halloysite nanotubes and nanocellulose incorporated PVA-PEO films: For food packaging applications. Food Packag. Shelf Life.

[10] Gómez-Estaca J., López-De-Dicastillo C., Hernández-Muñoz P., Catalá R., Gavara R. (2013). Advances in antioxidant active food packaging. Trends Food Sci. Technol..

[11] Yaqoob N., Zahira A., Kamal S., Almas M., Rehman S. (2023). Development of Multifunctional Bioactive Food Packaging Based on Silver Nanoparticles/Grape Fruit Peel Extract Reinforced PVA Composites. Mater. Today Commun..

[12] Gómez-Bachar L., Vilcovsky M., González-Seligra P., Famá L. (2024). Effects of PVA and yerba mate extract on extruded films of carboxymethyl cassava starch/PVA blends for antioxidant and mechanically resistant food packaging. Int. J. Biol. Macromol..

[13] Oun A.A., Shin G.H., Kim J.T. (2022). Antimicrobial, antioxidant, and pH-sensitive polyvinyl alcohol/chitosan-based composite films with aronia extract, cellulose nanocrystals, and grapefruit seed extract. Int. J. Biol. Macromol..

[14] Alshehri A.A., Hamed Y.S., Kamel R.M., Shawir S.M., Sakr H., Ali M., Ammar A., Saleh M.N., Fadly E.E., Salama M.A. (2023). Enhanced physical properties, antioxidant and antibacterial activity of bio-composite films composed from carboxymethyl cellulose and polyvinyl alcohol incorporated with broccoli sprout seed extract for butter packaging. Int. J. Biol. Macromol..

[15] Roy S., Rhim J.-W. (2021). Fabrication of bioactive binary composite film based on gelatin/chitosan incorporated with cinnamon essential oil and rutin. Colloids Surf. B Biointerfaces.

[16] Xu L., Xu X., Xu Y., Huang M. (2023). Oregano essential oil-doped citric acid modified polyvinyl alcohol bio-active films: Properties, bio-functional performance and active packaging application of chicken breast. Food Packag. Shelf Life.

[17] Rafique H.A.R., Lee B.-K. (2025). Bio-derived carbon dot-loaded PVA/CNC films with antioxidant, UV-blocking, and antimicrobial properties for active food packaging. Int. J. Biol. Macromol..

[18] Al-Tayyar N.A., Youssef A.M., Al-Hindi R. (2020). Antimicrobial food packaging based on sustainable Bio-based materials for reducing foodborne Pathogens: A review. Food Chem..

[19] Zhang W., Roy S., Ezati P., Yang D.-P., Rhim J.-W. (2023). Tannic acid: A green crosslinker for biopolymer-based food packaging films. Trends Food Sci. Technol..

[20] Madihalli S., Masti S.P., Eelager M.P., Chougale R.B., Dalbanjan N.P., Kumar S.K.P. (2025). Sodium alginate/poly(vinyl alcohol) active films incorporated with Chrysanthemum leaves extract as an eco-friendly approach to extend the shelf life of green chilies. Int. J. Biol. Macromol..

[21] Jakobek L., Šeruga M., Medvidović-Kosanović M., Novak I. (2007). Antioxidant Activity and Polyphenols of Aronia in Comparison to other Berry Species. Agric. Conspec. Sci..

[22] Ćujić N., Kardum N., Šavikin K., Zdunić G., Janković T., Menković N. (2018). Potential of chokeberry (*Aronia melanocarpa* L.) as a therapeutic food. Handbook of Food Bioengineering.

[23] Long W., Lin Y., Lv C., Dong J., Lv M., Lou X. (2024). High-compatibility properties of Aronia melanocarpa extracts cross-linked chitosan/polyvinyl alcohol composite film for intelligent food packaging. Int. J. Biol. Macromol..

[24] Wu X., Yan X., Zhang J., Wu X., Luan M., Zhang Q. (2024). Preparation and characterization of pH-sensitive intelligent packaging films based on cassava starch/polyvinyl alcohol matrices containing Aronia melanocarpa anthocyanins. LWT.

[25] Jakobek L., Drenjančević M., Jukić V., Šeruga M. (2012). Phenolic acids, flavonols, anthocyanins and antiradical activity of “Nero”, “Viking”, “Galicianka” and wild chokeberries. Sci. Hortic..

[26] Ferreira C., Ribeiro C., Nunes F.M. (2024). Effect of storage conditions on phenolic composition, vitamin C and antioxidant activity of ‘Golden Delicious’ and ‘Red Delicious’ apples. Postharvest Biol. Technol..

[27] Waseem M., Naz F., Waqas M., Jawaid M., Iqbal S.Z., Ali S. (2025). Characterization and application of chitosan, lignin, and fruit peel extracts based edible coatings for the preservation of apple fruits. Food Chem..

[28] Riaz A., Lei S., Akhtar H.M.S., Wan P., Chen D., Jabbar S., Abid M., Hashim M.M., Zeng X. (2018). Preparation and characterization of chitosan-based antimicrobial active food packaging film incorporated with apple peel polyphenols. Int. J. Biol. Macromol..

[29] Van Nguyen S., Lee B.-K. (2022). Multifunctional nanocomposite based on polyvinyl alcohol, cellulose nanocrystals, titanium dioxide, and apple peel extract for food packaging. Int. J. Biol. Macromol..

[30] Tian R., Yang W., Xue Q., Gao L., Huo J., Ren D., Chen X. (2015). Rutin ameliorates diabetic neuropathy by lowering plasma glucose and decreasing oxidative stress via Nrf2 signaling pathway in rats. Eur. J. Pharmacol..

[31] Gullón B., Lú-Chau T.A., Moreira M.T., Lema J.M., Eibes G. (2017). Rutin: A review on extraction, identification and purification methods, biological activities and approaches to enhance its bioavailability. Trends Food Sci. Technol..

[32] Rashidinejad A., Dima C., Karaca A.C., Jafari S.M. (2024). A review on rutin-loaded nanocarriers: Fundamentals, bioavailability, application in functional foods, and challenges. Eur. Polym. J..

[33] Narasagoudr S.S., Hegde V.G., Chougale R.B., Masti S.P., Vootla S., Malabadi R.B. (2020). Physico-chemical and functional properties of rutin induced chitosan/poly(vinyl alcohol) bioactive films for food packaging applications. Food Hydrocoll..

[34] Jakobek L., Boc M., Barron A.R. (2015). Optimization of Ultrasonic-Assisted Extraction of Phenolic Compounds from Apples. Food Anal. Methods.

[35] Hermans P.H., Weidinger A. (1949). X-ray Studies on the Crystallinity of Cellulose. J. Mater. Sci..

[36] Rabiej S.A. (1991). Comparison of Two X-Ray Diffraction Procedures for Crystallinity Determination. Eur. Polym. J..

[37] Ricciardi R., Gaillet C., Ducouret G., Lafuma F., Lauprêtre F. (2003). Investigation of the Relationships between the Chain Organization and Rheological Properties of Atactic Poly(Vinyl Alcohol) Hydrogels. Polymer.

[38] Hodge R.M., Edward G.H., Simon G.P. (1996). Water Absorption and States of Water in Semicrystalline Poly(Vinyl Alcohol) Films. Polymer.

[39] Sobral P.J.A., Menegalli F.C., Hubinger M.D., Roques M.A. (2001). Mechanical, water vapor barrier and thermal properties of gelatin based edible films. Food Hydrocoll..

[40] (2012). Grease Resistance Test for Paper and Paperboard.

[41] Gómez-Estaca J., Montero P., Fernández-Martín F., Alemán A., Gómez-Guillén M.C. (2008). Physical and chemical properties of tuna-skin and bovine-hide gelatin films with added aqueous oregano and rosemary extracts. Food Hydrocoll..

[42] Tongnuanchan P., Benjakul S., Prodpran T. (2012). Properties and antioxidant activity of fish skin gelatin film incorporated with citrus essential oils. Food Chem..

[43] (2021). Microbiology of the Food Chain—Horizontal Method for the Enumeration of Coagulase-Positive Staphylococci (Staphylococcus aureus and Other Species), Part 1: Method Using Baird-Parker Agar Medium–2.

[44] Costa P., Lobo J.M.S. (2001). Modeling and Comparison of Dissolution Profiles. Eur. J. Pharm. Sci..

[45] Dash S., Murthy P.N., Nath L., Chowdhury P. (2010). Kinetic modeling on drug release from controlled drug delivery systems. Acta Pol. Pharm..

[46] Higuchi T. (1963). Mechanism of Sustained-action Medication. Theoretical Analysis of Rate of Release of Solid Drugs Dispersed in Solid Matrices. J. Pharm. Sci..

[47] Korsmeyer R.W., Gurny R., Doelker E., Buri P., Peppas N.A. (1983). Mechanisms of Solute Release from Porous Hydrophilic Polymers. Int. J. Pharm..

[48] Peppas N.A. (1985). Analysis of Fickian and non-Fickian drug release from polymers. Pharm. Acta Helv..

[49] Pignitter M., Somoza V. (2012). Critical evaluation of methods for the measurement of oxidative rancidity in vegetable oils. J. Food Drug Anal..

[50] Li Z., Niu X., Cao Y., Tang S., Zhang Y., Hai J., Feng X., Li M.T., Li J., Zhong T. (2026). Chitosan and chitosan derivative-based composite packaging for food preservation: Preparation strategies, performance modulation, and application prospects. Int. J. Biol. Macromol..

[51] Talebi A., Esmaiili M., Almasi H., Hamishehkar H. (2026). Development of smart/active chitosan/Kapppa-carrageenan films incorporating Aronia anthocyanin and ginger essential oil Pickering emulsion stabilized by hempseed protein nanoparticles. Food Chem..

[52] Uma K., Manjunath C., Pawar S., Anupama C. (2024). Biopolymer-TiO2 antibacterial nanocomposite films as food packaging materials. Results Mater..

[53] Koshy R.R., Vishnu K., Reghunadhan A., Mary S.K., Koshy J.T., Sangeetha D., Williams P.G., Pothan L.A. (2024). Biofilms from poly-vinyl alcohol/palmyra root sprout with Boswellia serrata, carbon dots and anthocyanin for sensing the freshness of sardine fish. Int. J. Biol. Macromol..

[54] Talib H.M., Yousif A.A., Alkhayatt A.H.O. (2024). The impact of thiourea concentration on the Structural, surface morphologyand optical characterization of chemically deposited CdS-PVA nanocomposites thin films. Results Opt..

[55] Ghaderi J., Hosseini S.F., Keyvani N., Gómez-Guillén M.C. (2019). Polymer blending effects on the physicochemical and structural features of the chitosan/poly(vinyl alcohol)/fish gelatin ternary biodegradable films. Food Hydrocoll..

[56] Franca T., Goncalves D., Cena C. (2022). ATR-FTIR spectroscopy combined with machine learning for classification of PVA/PVP blends in low concentration. Vib. Spectrosc..

[57] Zhang C., Sun G., Cao L., Wang L. (2020). Accurately intelligent film made from sodium carboxymethyl starch/κ-carrageenan reinforced by mulberry anthocyanins as an indicator. Food Hydrocoll..

[58] Ma Q., Liang T., Cao L., Wang L. (2017). Intelligent poly(vinyl alcohol)-chitosan nanoparticles-mulberry extracts films capable of monitoring pH variations. Int. J. Biol. Macromol..

[59] Song J., Yang W., Zhao X., Chen S., Qian G., Jiang G., Ren S., Li S. (2021). Composite films with excellent mechanical, antioxidant and UV-shielding properties prepared from oligomeric proanthocyanidin nanospheres and poly(vinyl alcohol). Ind. Crops Prod..

[60] Jiang S., Qiao C., Liu R., Liu Q., Xu J., Yao J. (2023). Structure and properties of citric acid cross-linked chitosan/poly(vinyl alcohol) composite films for food packaging applications. Carbohydr. Polym..

[61] Hosseini S.F., Nahvi Z., Zandi M. (2018). Antioxidant peptide-loaded electrospun chitosan/poly(vinyl alcohol) nanofibrous mat intended for food biopackaging purposes. Food Hydrocoll..

[62] Zhang W., Li Z., Jin W., Xu Y., Mei F., Cheng D., Cai G., Wang X. (2025). Biomass-based food packaging film of chitosan/flavonoids/silver nanoparticle for shelf life extension of food. Colloids Surf. A Physicochem. Eng. Asp..

[63] Li X., Fan Y., Guo J., Li R., Liu Z., Hou Y., Qu Z., Liu Q. (2024). Polyvinyl alcohol/kappa-carrageenan-based package film with simultaneous incorporation of ferric ion and polyphenols from Capsicum annuum leaves for fruit shelf-life extension. Int. J. Biol. Macromol..

[64] Almazrouei M., Adeyemi I., Janajreh I. (2022). Thermogravimetric assessment of the thermal degradation during combustion of crude and pure glycerol, Biomass Convers. Biorefin.

[65] Vu N.Q., Le T.M., Ngo A.N.B., Nguyen M.H.T., Liao Y.-C., Tran T.T. (2025). Engineering Functional PVA: A comprehensive review of chemical modifications and prospective developments. ACS Polym. Au.

[66] Bonilla J., Fortunati E., Atarés L., Chiralt A., Kenny J.M. (2013). Physical, structural and antimicrobial properties of poly vinyl alcohol–chitosan biodegradable films. Food Hydrocoll..

[67] Koosha M., Hamedi S. (2018). Intelligent Chitosan/PVA nanocomposite films containing black carrot anthocyanin and bentonite nanoclays with improved mechanical, thermal and antibacterial properties. Prog. Org. Coat..

[68] Yong H., Liu J., Qin Y., Bai R., Zhang X., Liu J. (2019). Antioxidant and pH-sensitive films developed by incorporating purple and black rice extracts into chitosan matrix. Int. J. Biol. Macromol..

[69] Qin Y., Xu F., Yuan L., Hu H., Yao X., Liu J. (2020). Comparison of the physical and functional properties of starch/polyvinyl alcohol films containing anthocyanins and/or betacyanins. Int. J. Biol. Macromol..

[70] Peter A., Pop L., Cozmuta L.M., Nicula C., Cozmuta A.M., Drazic G., Magyari K., Muresan-Pop M., Todea M., Baia L. (2024). Beeswax-poly(vinyl alcohol) composite films for bread packaging. Food Chem. X.

[71] Zhang K., Huang T.-S., Yan H., Hu X., Ren T. (2019). Novel pH-sensitive films based on starch/polyvinyl alcohol and food anthocyanins as a visual indicator of shrimp deterioration. Int. J. Biol. Macromol..

[72] Zhang S., Zhao H. (2017). Preparation and properties of zein–rutin composite nanoparticle/corn starch films. Carbohydr. Polym..

[73] Hasan M., Utami A., Siregar C., Hanum L., Khaldun I., Nazar M. (2025). Thermal, Structural and Mechanical Characterization of Bio-Nanocomposite Films from Purple Sweet Potato Starch/Chitosan Nanoparticles Integrated with Anthocyanin Extract. Results Eng..

[74] Eranda D.H.U., Chaijan M., Castro-Munoz R. (2024). Current advances in surface wettability in food packaging materials: Strategies, methods and future trends. J. Food Eng..

[75] Prietto L., Mirapalhete T.C., Pinto V.Z., Hoffmann J.F., Vanier N.L., Lim L., Dias A.R.G., Da Rosa Zavareze E. (2017). PH-sensitive films containing anthocyanins extracted from black bean seed coat and red cabbage. LWT.

[76] Sun L., Sun J., Chen L., Niu P., Yang X., Guo Y. (2017). Preparation and characterization of chitosan film incorporated with thinned young apple polyphenols as an active packaging material. Carbohydr. Polym..

[77] Arora R., Haleem R., Agarwal A., Shukla S.K. (2025). Development and characterization of pea hull reinforced polyvinyl alcohol film for active packaging of chicken meatballs. Food Chem..

[78] Yong H., Liu J. (2020). Recent advances in the preparation, physical and functional properties, and applications of anthocyanins-based active and intelligent packaging film. Food Packag. Shelf Life.

[79] Khan M.R., Di Giuseppe F.A., Torrieri E., Sadiq M.B. (2021). Recent advances in biopolymeric antioxidant films and coatings for preservation of nutritional quality of minimally processed fruits and vegetables. Food Packag. Shelf Life.

[80] Juwita S., Indrawati A., Damajanti R., Safika S., Mayasari N.L.P.I. (2022). Genetic relationship of *Staphylococcus aureus* isolated from humans, animals, environment, and Dangke products in dairy farms of South Sulawesi Province, Indonesia. Vet. World.

[81] Amin M.U., Khurram M., Khattak B., Khan J. (2015). Antibiotic additive and synergistic action of rutin, morin and quercetin against methicillin resistant *Staphylococcus aureus*. BMC Complement. Altern. Med..

[82] Horvacki N.M., Milinčić D.D., Jović M.D., Dramićanin A.M., Fotirić-Akšić M.M., Pešić M.B., Milojković-Opsenica D.M. (2024). Bioassay-guided evaluation of antimicrobial properties and profile of bioactive compounds from leaf, peel and mesocarp of four apple cultivars (*Malus domestica* Borkh.) grown in Serbia: Application of HPTLC-EDA and UHPLC Q-ToF MS techniques. Food Chem..

[83] Gangadharan G., Puttaiahgowda Y.M., Gupta S., Kulal A. (2025). Antimicrobial and biodegradable PVA/rutin/CuO nanocomposite film for garlic preservation. Sci. Rep..

[84] Zhang L., Guo K.-J., Huang Y.-K., Zeng D.-P., Chen Y., Qiao D., Shaukat M., Qian J.-Y. (2025). Preparation and characterization of liposoluble tea polyphenol based functional emulsion films and the regulation of its releasing property. Food Chem..

[85] Wang S., Xia P., Wang S., Liang J., Sun Y., Yue P., Gao X. (2019). Packaging films formulated with gelatin and anthocyanins nanocomplexes: Physical properties, antioxidant activity and its application for olive oil protection. Food Hydrocoll..

