# Supplementary material for: Structure–Property Relationships in Bioactive Poly (Vinyl Alcohol) Films Incorporating Natural Phenolic Additives for Sustainable Active Packaging
Source: Polymers (Basel). 2026 Oct 14;18(20):2519. doi: 10.3390/polym18202519 (PMC13708831; doi:10.3390/polym18202519)
Supplement: Supplementary file 1 [file polymers-18-02519-s001.zip › polymers-4588554-supplementary.pdf]

Structure–Property Relationships in Bioactive Poly (vinyl alcohol) Films Incorporating Natural Phenolic Additives for Sustainable Active Packaging

Supplementary material

Table S1. The O/C ratio from the SEM analysis.

	WT %								AT %		RAPORT
	C	O	K	Ca	Mg	P	Si	Cl	C	O	O/C
PVA	60.1	39.9							5.008333	2.49375	0.49792
PVA + AP	56.2	43.4	0.3					0.1	4.683333	2.7125	0.579181
PVA + R	57.7	42.3							4.808333	2.64375	0.549827
PVA + A	49.5	49.6	0.6	0.1	0.1				4.125	3.1	0.751515

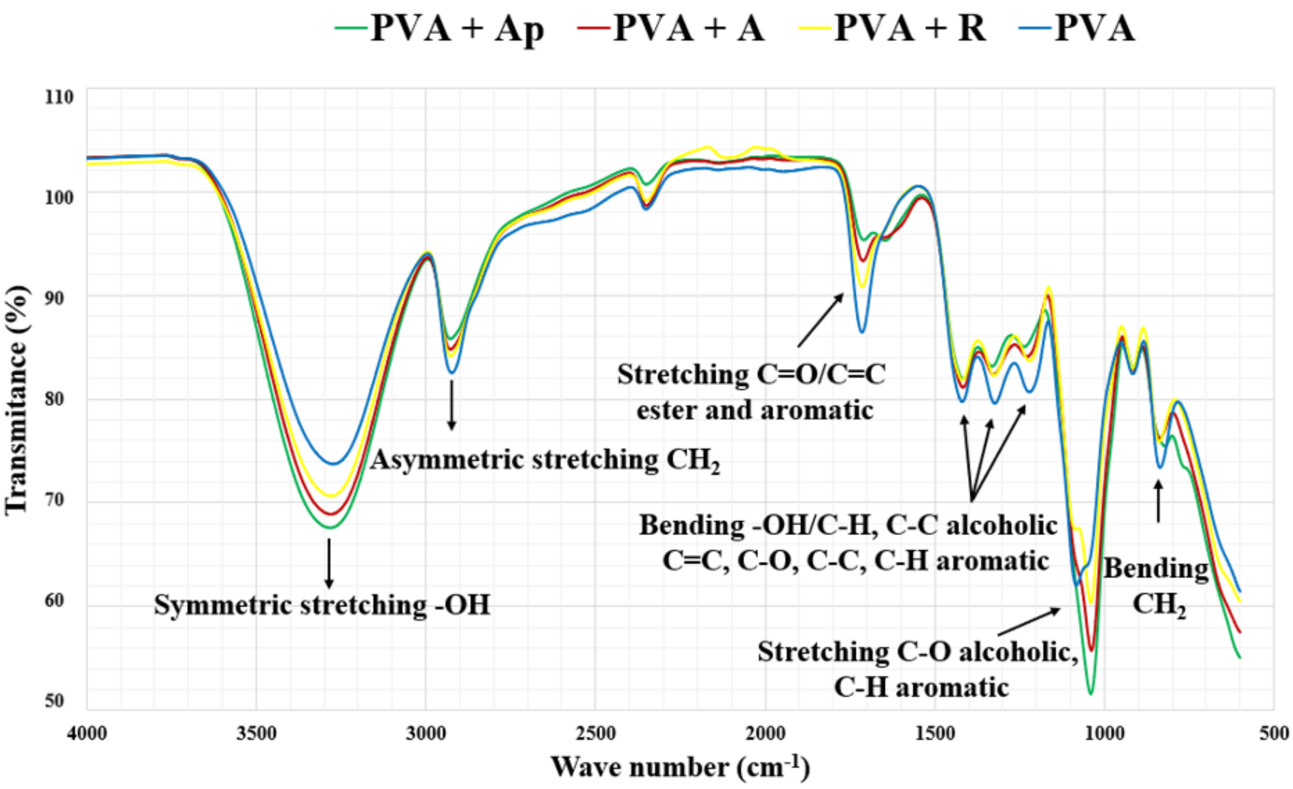

Figure S1. FTIR spectra of films produced.

Table S2. Data extracted from the deconvolution of XRD spectra.

Sample / Formulation	$xc_{\text{crystalline}}$ (o)	A_c (Crystalline Area)	A_a (Amorphous Area)	Crystallinity index (CI, %)	R^2	Structural state
PVA	-	0.00 ± 0.00	100.00 ± 0.00	0.00 ± 0.00 d	-	Completely amorphous
PVA + AP	19.93	2071.91 ± 22.61	9969.97 ± 69.37	17.21 ± 0.39 c	0.967	Semi-crystalline
PVA + R	19.92	2282.27 ± 19.31	9834.99 ± 65.26	18.83 ± 0.24 b	0.960	Semi-crystalline
PVA + A	20.07	2904.09 ± 26.29	11677.10 ± 81.65	19.92 ± 0.48 a	0.969	Semi-crystalline
R - powder	19.88	2187.64 ± 21.01	8928.24 ± 96.54	19.68 ± 0.41	0.961	Semi-crystalline
A - powder	-	0.00 ± 0.00	100.00 ± 0.00	0.00 ± 0.00	-	Completely amorphous
AP - powder	-	0.00 ± 0.00	100.00 ± 0.00	0.00 ± 0.00	-	Completely amorphous

Section S1. Quantitative Determination of the Crystallinity Index (CI, %) by XRD Deconvolution

To quantitatively evaluate the relative degree of crystallinity, the X-ray diffraction (XRD) profiles of PVA and bioactive composite films were deconvoluted using OriginPro 9.0 software (OriginLab Corporation, Northampton, MA, USA) with the Peak Analyzer tool, following the general peak deconvolution approach proposed by Hermans and Weidinger [35, 36].

Prior to deconvolution, the diffractograms were restricted to the angular range of $2\theta = 10° - 45°$, which encompasses all characteristic reflections and the broad amorphous scattering, while excluding high-angle instrumental background noise. A constant horizontal baseline was established corresponding to the minimum background intensity.

The diffraction profiles were fitted using non-linear least-squares curve fitting with Gaussian peak functions. The deconvolution model comprised two primary components:

1. A sharp crystalline reflection centered at $2\theta \approx 19.5° \pm 0.1°$, assigned to the characteristic $(10\bar{1})$ crystallographic plane of semi-crystalline PVA [37, 38].
2. A broad amorphous halo centered at $2\theta \approx 21.5° - 23.5°$, representing the disordered polymer phase. To ensure physical consistency and prevent artificial peak narrowing, the full width at half maximum (FWHM, w) of the amorphous halo was constrained ($w > 6.0°$).

Iterations were performed until convergence was achieved, with fit coefficients (R^2, COD) exceeding 0.95 in all cases. The relative degree of crystallinity (crystallinity index, CI) was quantitatively calculated from the resolved peak areas according to the following equation [35, 36]:

$$CI(\%) = \frac{A_c}{A_c + A_a} \times 100$$

where

- A_c represents the integrated area of the crystalline diffraction peak(s) ($2\theta \approx 19.5°$);
- A_a denotes the integrated area of the broad amorphous halo.

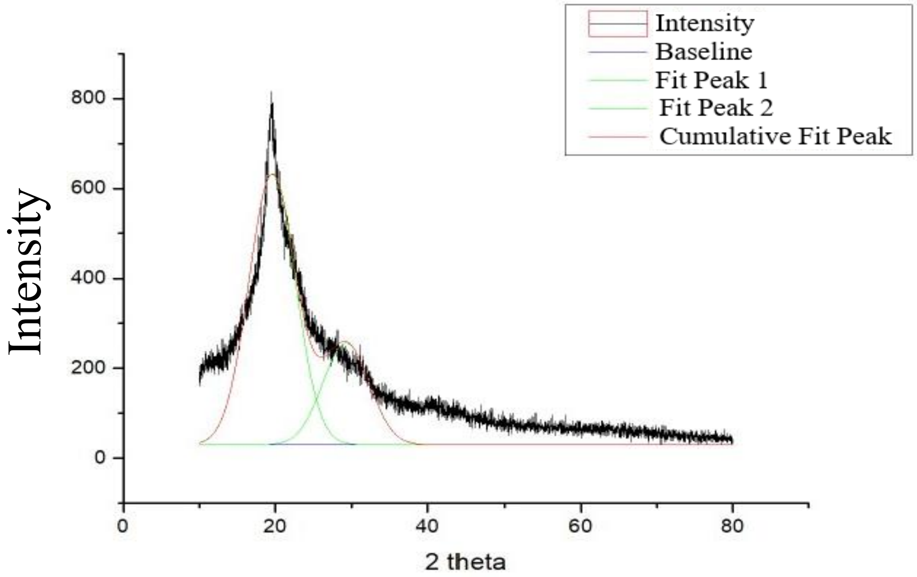

Figure S2. Deconvolution model XRD.

[35]. Hermans, P.H.; Weidinger, A. X-ray Studies on the Crystallinity of Cellulose. Journal of Polymer Science 1949, 4, 135–144, doi:10.1002/pol.1949.120040203.

[36]. Rabiej, S. A Comparison of Two X-Ray Diffraction Procedures for Crystallinity Determination. European Polymer Journal 1991, 27, 947–954, doi:10.1016/0014-3057(91)90038-p.

[37]. Ricciardi, R.; Gaillet, C.; Ducouret, G.; Lafuma, F.; Lauprêtre, F. Investigation of the Relationships between the Chain Organization and Rheological Properties of Atactic Poly(Vinyl Alcohol) Hydrogels. Polymer 2003, 44, 3375–3380, doi:10.1016/s0032-3861(03)00246-5.

[38]. Hodge, R.M.; Edward, G.H.; Simon, G.P. Water Absorption and States of Water in Semicrystalline Poly(Vinyl Alcohol) Films. Polymer 1996, 37, 1371–1376, doi:10.1016/0032-3861(96)81134-7.

Table S3. Deconvolution C1s.

sample	C-C/C-H	%	C-OH C-O-C C-N	%	C=O	%	O-C=O	%
PVA	284.6	84.4	286.1	10.3	287.9	4.3	289	1
Apple	284.6	64.8	286.1	28.4	287.6	5.4	289.1	1.4
PVA_apple	284.6	24.5	286.1	59.8	287.5	11	288.8	4.7
Aronia	284.6	44.7	286.1	45.4	288	4.9	289.9	5
PVA_aronia	284.6	47.7	286.1	39.9	287.5	6.4	288.8	6
Rutin	284.6	26.1	286	50.2	287.1	19.9	289.9	3.8
PVA_rutin	284.6	67	286.1	25.7	287.2	3.1	288.9	4.2

Table S4. PV (meq/kg) recorded by butter packaged or unpackaged. Where A—aronia; AP—apple peel; R—rutin; C. PACK.—commercial packaging; UNPACK.—without packaging.

	DAY 4	DAY 14	DAY 43	DAY 77
PVA+A	1.19	1.21	2.03	2.72
PVA+R	1.19	1.20	2.23	2.80
PVA+AP	1.20	1.22	2.57	2.98
C. PACK.	1.21	1.22	2.46	3.32
PVA	1.21	1.27	2.84	3.64
UNPACK.	1.22	1.50	2.90	3.91

Section S2. Kinetics of polyphenol release

Non-linear least-squares regression was employed to analyze the cumulative release data as a function of immersion time (t). Given that a substantial fraction of the total polyphenol content ($> 86\%$) was discharged within the initial 5 min, the release profiles across the entire timeline (0–120 min) were fitted to the asymptotic first-order kinetic model [44-46]:

$$C_t = C_{\text{max calc.}} \cdot (1 - e^{-k_1 \cdot t})$$

where

C_t is the cumulative concentration of polyphenols released at time t (µg GAE/g film); $C_{\text{max calc.}}$ represents the theoretical asymptotic maximum concentration released at equilibrium / infinite time (µg GAE/g film); k_1 is the first-order release rate constant (min^{-1}), reflecting the velocity of the initial discharge burst driven by the steep concentration gradient; t is the release time (min).

Furthermore, because the extensive initial burst release ($> 86\%$ at $t = 5$ min) exceeded the valid theoretical boundary condition ($M_t/M_\infty \leq 0.60$) required for the classical power-law Korsmeyer–Peppas model [47, 48], the diffusion-controlled mechanism within the cross-linked polymer network was evaluated specifically for the secondary slow-release phase (5-120 min). For this plateau region, the release data were fitted to the modified Higuchi model [46]:

where

C_t is the cumulative polyphenol concentration at time t ($t \in [5{,}120]$ min); C_0 is the intercept (µg GAE/g film), denoting the baseline concentration established immediately following the initial rapid surface burst at 5 min; k_H is the Higuchi dissolution constant (µg · g^{-1} · $\text{min}^{-0.5}$), reflecting the rate of pure Fickian diffusion of entrapped polyphenols migrating through the tortuous PVA network.

The goodness of fit for each mathematical model was assessed using the coefficient of determination (R^2, COD), adjusted R^2 (Adj. R^2), and the reduced chi-square (χ^2_{red}) values.

[44]. Costa, P.; Lobo, J.M.S. Modeling and Comparison of Dissolution Profiles. European Journal of Pharmaceutical Sciences 2001, 13, 123–133, doi:10.1016/s0928-0987(01)00095-1.

[45]. Dash, S., Murthy, P. N., Nath, L., & Chowdhury, P. (2010). Kinetic modeling on drug release from controlled drug delivery systems. Acta Poloniae Pharmaceutica - Drug Research, 67(3), 217–223.

[46]. Higuchi, T. Mechanism of Sustained-action Medication. Theoretical Analysis of Rate of Release of Solid Drugs Dispersed in Solid Matrices. Journal of Pharmaceutical Sciences 1963, 52, 1145–1149, doi:10.1002/jps.2600521210.

[47]. Korsmeyer, R.W.; Gurny, R.; Doelker, E.; Buri, P.; Peppas, N.A. Mechanisms of Solute Release from Porous Hydrophilic Polymers. International Journal of Pharmaceutics 1983, 15, 25–35, doi:10.1016/0378-5173(83)90064-9.

[48]. Peppas, N. A. (1985). Analysis of Fickian and non-Fickian drug release from polymers. Pharmaceutica Acta Helvetiae, 60(4), 110–111.

The graphics are set according to the kinetic model of the first order.

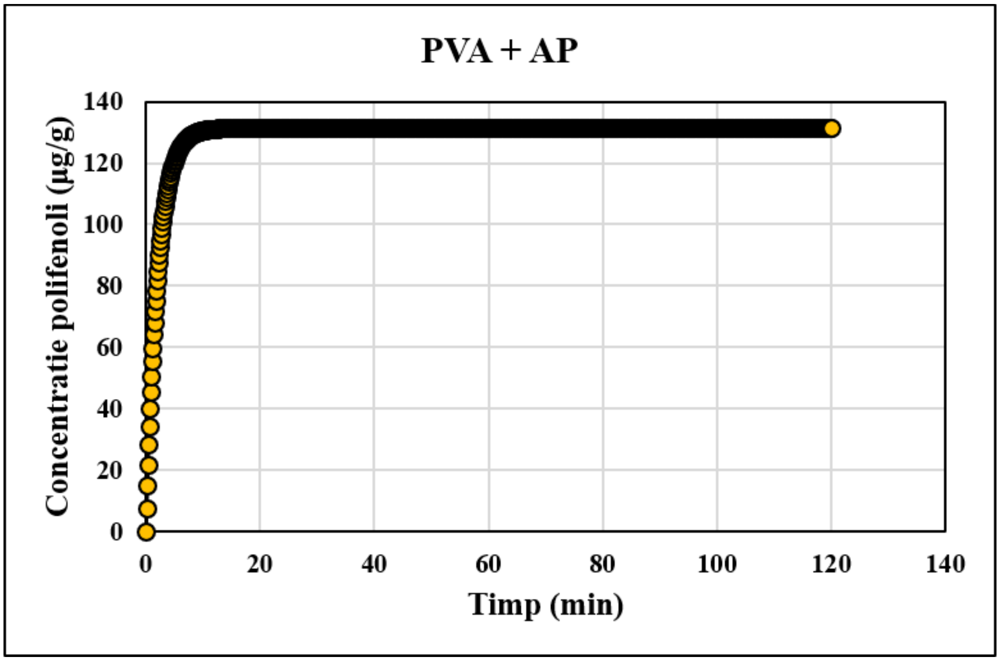

Figure S3. The graphics are set according to the kinetic model of the first order, PVA + AP.

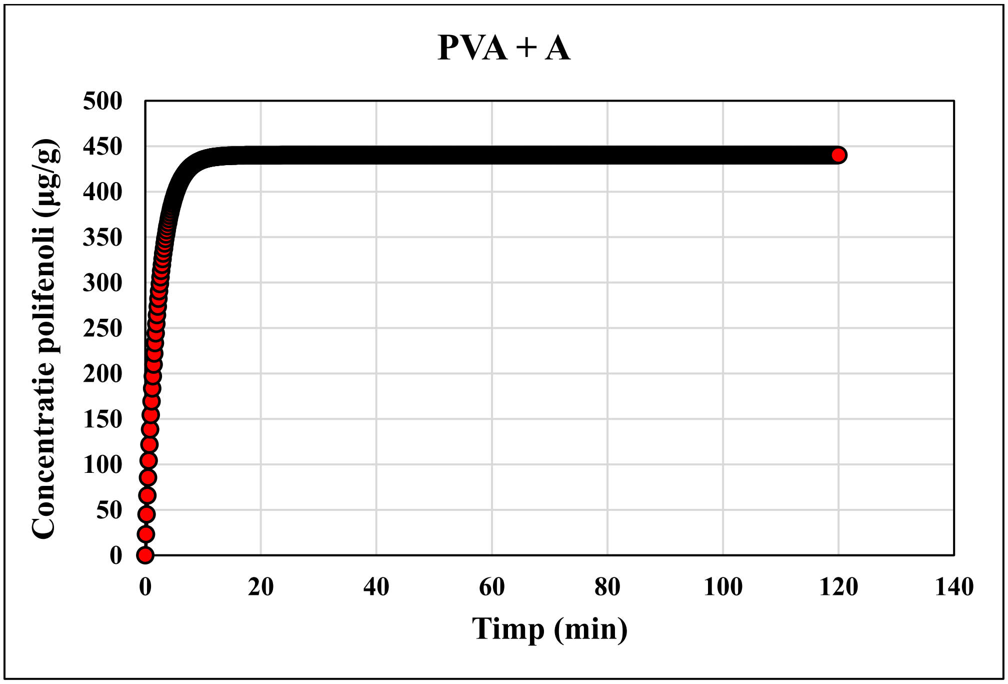

Figure S4. The graphics are set according to the kinetic model of the first order, PVA + A.

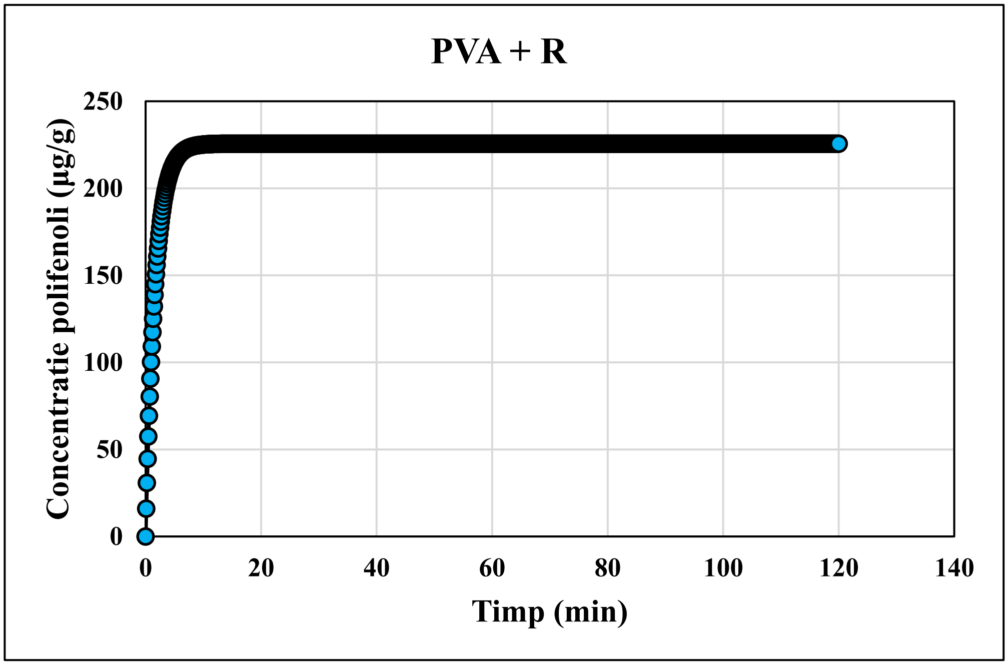

Figure S5. The graphics are set according to the kinetic model of the first order, PVA + R.
